# The earliest Pleistocene record of a large-bodied hominin from the Levant supports two out-of-Africa dispersal events

**DOI:** 10.1038/s41598-022-05712-y

**Published:** 2022-02-02

**Authors:** Alon Barash, Miriam Belmaker, Markus Bastir, Michalle Soudack, Haley D. O’Brien, Holly Woodward, Amy Prendergast, Omry Barzilai, Ella Been

**Affiliations:** 1 Azrieli Faculty of Medicine, Bar Ilan University, POB 1589, 1311502 Safed, Israel; 2 The Department of Anthropology, The University of Tulsa, Tulsa, OK 74104 USA; 3 Paleoanthropology Group, Department of Paleobiology, Museo Nacional de Ciencias Naturales, CSIC, JG Abascal 2, 28006 Madrid, Spain; 4 Sackler Faculty of Medicine, Tel Aviv University, Tel Aviv, Israel; 5 Department of Pediatric Imaging, The Edmond and Lily Safra Children’s Hospital, Chaim Sheba Medical Center, Tel Hashomer, Ramat Gan, Israel; 6 Department of Anatomy and Cell Biology, Oklahoma State University Center for Health Science, Tulsa, USA; 7 School of Geography, Earth and Atmospheric Sciences, University of Melbourne, Melbourne, Australia; 8 Archaeological Research Department, Israel Antiquities Authority, POB 586, 91004 Jerusalem, Israel; 9 Department of Sports Therapy, Faculty of Health Professions, Ono Academic College, Kiryat Ono, Israel; 10 Department of Anatomy and Anthropology, Sackler Faculty of Medicine, Tel-Aviv University, Tel-Aviv, Israel

**Keywords:** Biological anthropology, Behavioural ecology

## Abstract

The paucity of early Pleistocene hominin fossils in Eurasia hinders an in-depth discussion on their paleobiology and paleoecology. Here we report on the earliest large-bodied hominin remains from the Levantine corridor: a juvenile vertebra (UB 10749) from the early Pleistocene site of ‘Ubeidiya, Israel, discovered during a reanalysis of the faunal remains. UB 10749 is a complete lower lumbar vertebral body, with morphological characteristics consistent with *Homo* sp. Our analysis indicates that UB-10749 was a 6- to 12-year-old child at death, displaying delayed ossification pattern compared with modern humans. Its predicted adult size is comparable to other early Pleistocene large-bodied hominins from Africa. Paleobiological differences between UB 10749 and other early Eurasian hominins supports at least two distinct out-of-Africa dispersal events. This observation corresponds with variants of lithic traditions (Oldowan; Acheulian) as well as various ecological niches across early Pleistocene sites in Eurasia.

## Introduction

**Figure 1:**
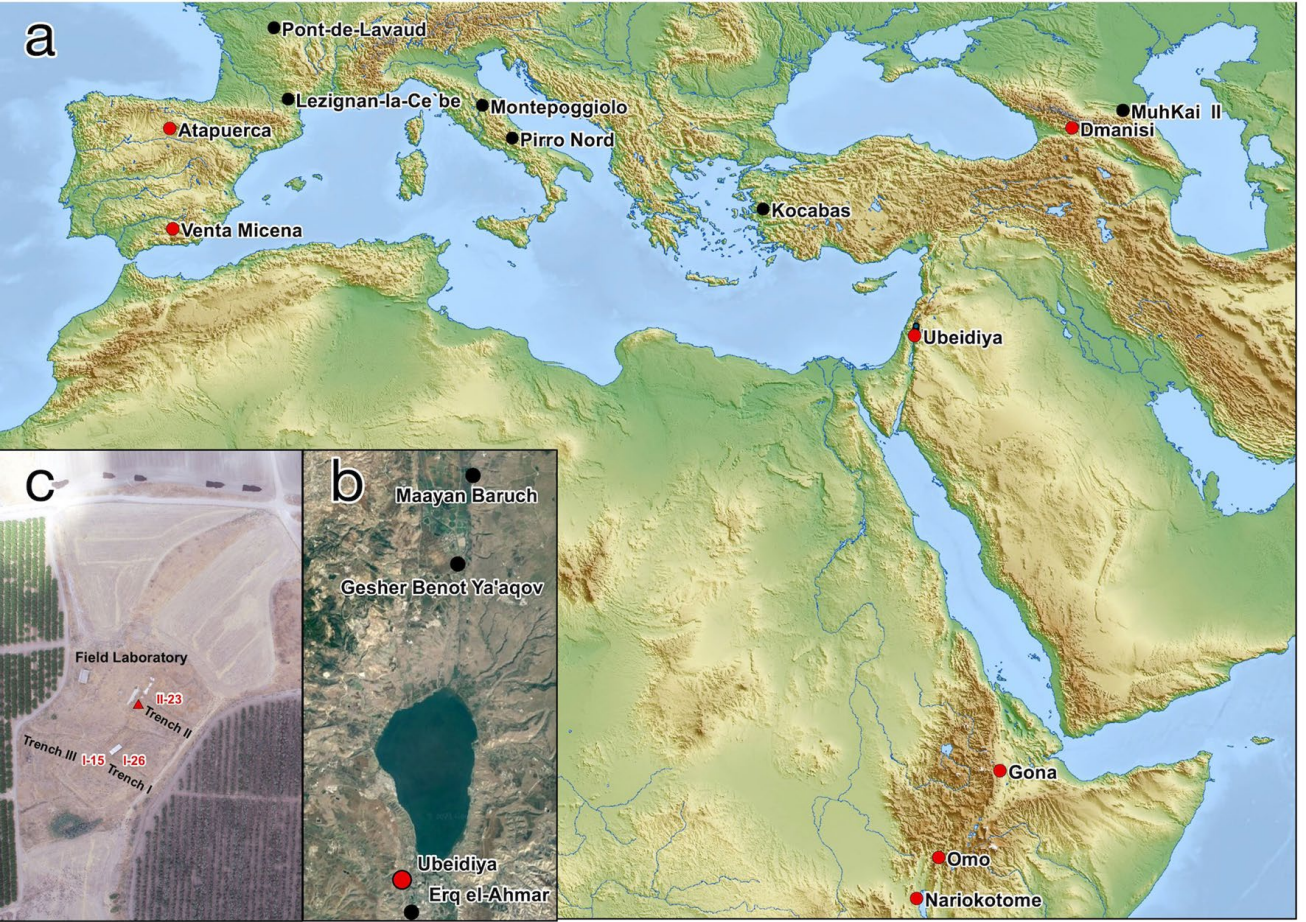
‘Ubeidya site locality. (**a**) Map of Africa and Eurasia with major Pleistocene paleoanthropological sites. Black circles denote sites with no osteological remains; red circles denote sites with human osteological remains. (**b**) The location of the site of ‘Ubeidiya, south of lake Kineret (Sea of Galilee), on the western banks of the Jordan Valley (red circle) (**c**) aerial photograph of the excavation plan of ‘Ubeidiya with the location of layer II-23 where UB 10749 was recovered.

The Levant region, the major land bridge connecting Africa with Eurasia, was a significant dispersal route for Hominins and fauna during the early Pleistocene^1–3^. But while there are numerous Eurasian early Pleistocene sites, fossil hominin remains are rare and present only at four localities dated between 1.1 and 1.9 Mya^4–11^: Dmanisi (Georgia), Venta Micena (Orce, Granada), Modjokerto and Sangiran (Java, Indonesia), and Sima De Elefante (Atapuerca, Spain) (Supplementary 2: Table 1; Fig. 1a). In contrast, early Pleistocene east African sites containing *Homo* cranial remains are more abundant, but postcranial remains are scarcer, and the best-preserved skeleton is Nariokotome KNM-WT 15000^12,13^.

In the Levant, the only site from this time-period with hominin remains is ‘Ubeidiya at the western escarpment of the Jordan Valley which is a part of the broader Rift Valley (Supplementary 1: Fig. 1b,c). The fossil remains include cranial fragments (UB 1703, 1704, 1705, and 1706), two incisor (UB 1700, UB 335) and a molar (UB 1701), identified as *Homo* cf. *erectus*/*ergaster*^14–18^. It is important to note that some of these fragments were bulldozed out of the ground preceding the first season, while others are considered intrusive and younger than the surroundings deposits^17^.

In 2018 during a reanalysis of the faunal assemblages done by two of the authors (A. B, and M. B.) a complete vertebral body (UB 10749) with hominin characteristics was found. This is the first hominin postcranial remain found at ‘Ubeidiya securely assigned to early Pleistocene deposits (See “Materials and methods”).

Here we assess the taxonomic affinity of UB 10749, its serial location along the spinal column, its chronological and physiological age at death, estimate the specimen's height and weight, and detect any pathological or taphonomic changes. Based on our findings, we explore the unique developmental characteristics of the UB 10749 within the context of early *Homo* paleobiology and its implications for hominin dispersal out of Africa.

### Description of the finding

**Figure 2:**
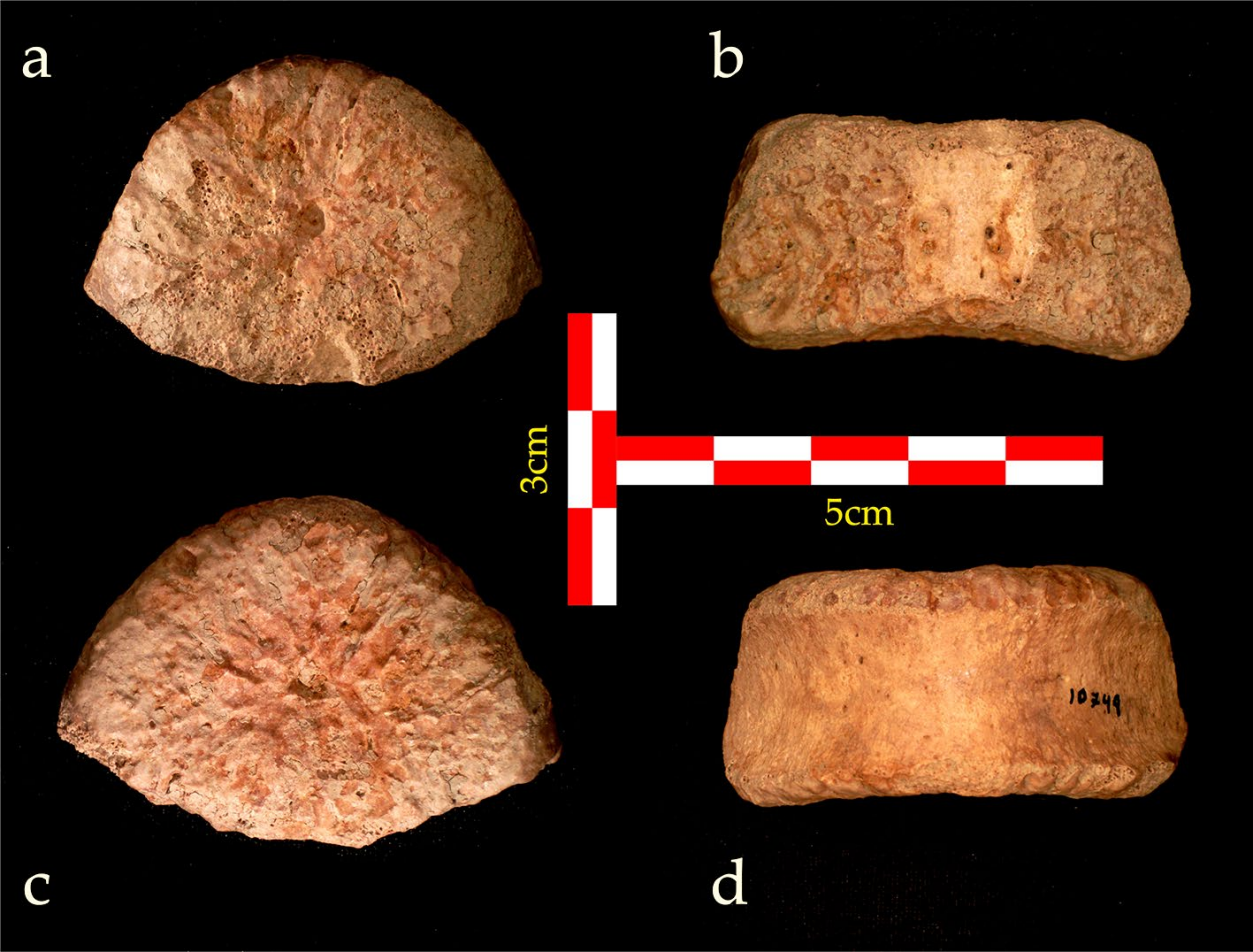
UB 10749 vertebral body. (**a**) Superior view; (**b**) posterior view; (**c**) inferior view; (**d**) anterior view.

UB 10749 is a complete vertebral body (Fig. 2). The superior plate of the vertebra is oval, with an uneven surface, indicating non-ossification of the vertebral endplate. Similarly, the inferior plate is also oval with marked postero-lateral edges. A small pit is found in the center of both superior and inferior plates. The inferior plate is bilaterally wider than the superior plate. The anterior and lateral walls are smooth and slightly concave i.e., their superior and inferior edges are more prominent than the center. There is no evidence of rib attachment to the body on the lateral walls. The posterior wall can be divided into three parts, the center and right and left lateral thirds. The central part is smooth with two nutritional foramina. The two lateral thirds are located at the junction between the vertebral body and the pedicles. Their surface is uneven, indicating that the pedicle had not yet ossified to the vertebral body. In a lateral view, the vertebra shows a lordotic wedging as the height of the anterior wall is greater than that of the posterior wall (Supplementary 2: Table 2). The oval shape of the vertebral body, the concavity of the inferior plate, the lordotic wedging, and the lack of rib bearing facets all indicate a lower lumbar vertebra, i.e., presacral (PS) 1, PS2, or PS3 (corresponding to L5, L4, and L3 in modern humans).

**Figure 3:**
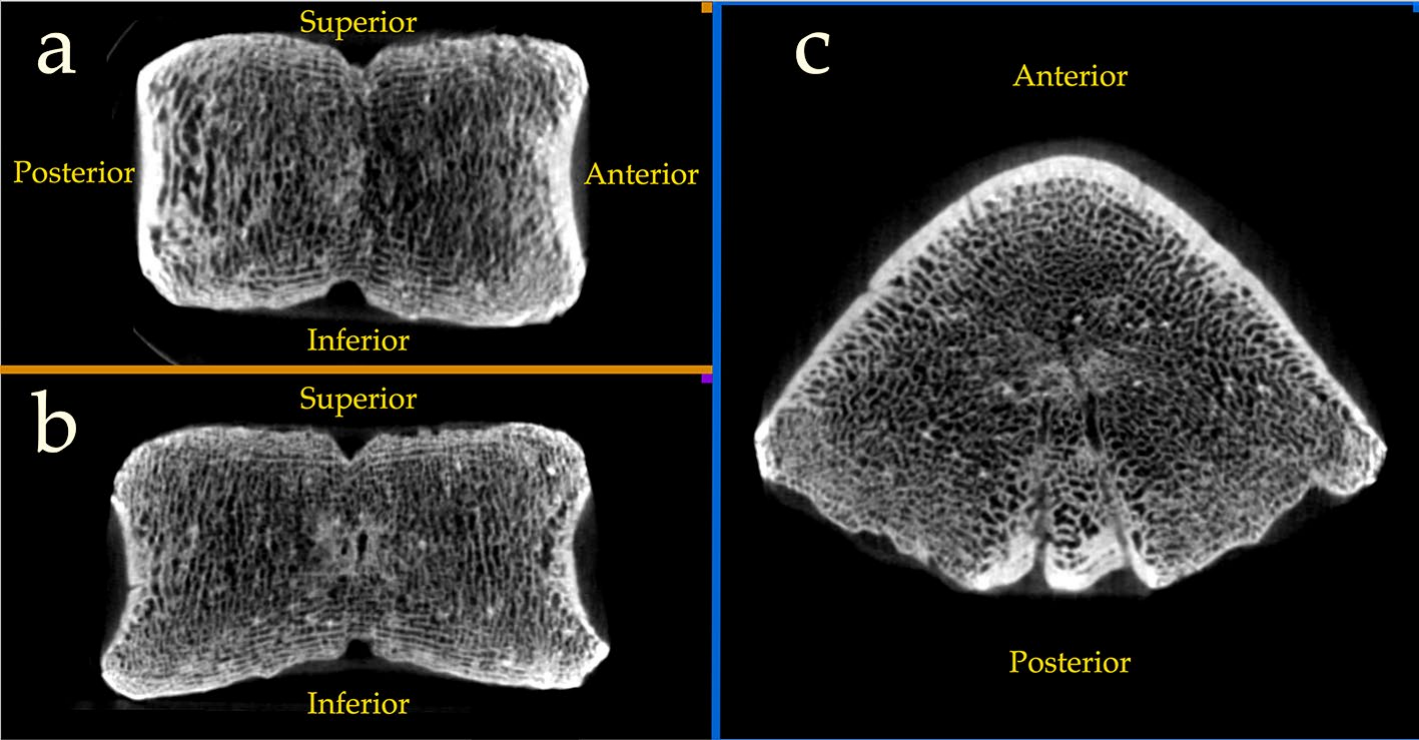
µCT scan of UB 10749. (**a**) Midsagittal section. (**b**) Coronal section. (**c**) Horizontal section.

A micro-CT (µCT) scan of UB 10749 (Fig. 3) reveals a well-developed cortical bone on the anterior and lateral walls and the central part of the posterior wall. The cancellous bone at the superior and inferior plates is very thin, as is the bone at the lateral thirds of the posterior wall, indicating that these were not yet ossified. The µCT scan also reveals well-developed canals within the vertebral body –Bastons' venous plexus^19^ (Fig. 3c). A small pit at the superior and inferior plates is seen in the mid-sagittal and coronal planes of the CT scan (Fig. 3a, b). A thin vertical region that appears black on the µCT connects the two pits, indicating that this area was not yet ossified.

### Taxonomic identification

We compared UB 10749 to a range of mammalian species from, but not limited to, those present in ‘Ubeidiya such as carnivores (e.g., *Ursus*, *Hyeana*, *Panthera*), Artiodactyla (e.g., *Hippopotamus, Praemegaceros*), Perissodactyla (Rhinocertidae, Equidae), Proboscidea (*Mamuthus*, *Elephas*), and Primates (*Homo*, *Pongo*, *Gorilla*, *Theropithecus* and *Papio*).

UB 10749 lacks the inward indentation on the posterior wall distinctive of *Ursus* and is short cranio-caudally, as opposed to the longer vertebral bodies of ungulates.

The size, the large vertebral plate, and the relatively short vertebral body of UB 10749 indicates that it belongs to hominoidea. The lordotic wedging and the concavity of the inferior plate further suggests that this is a hominin vertebra^20,21^.

**Figure 4:**
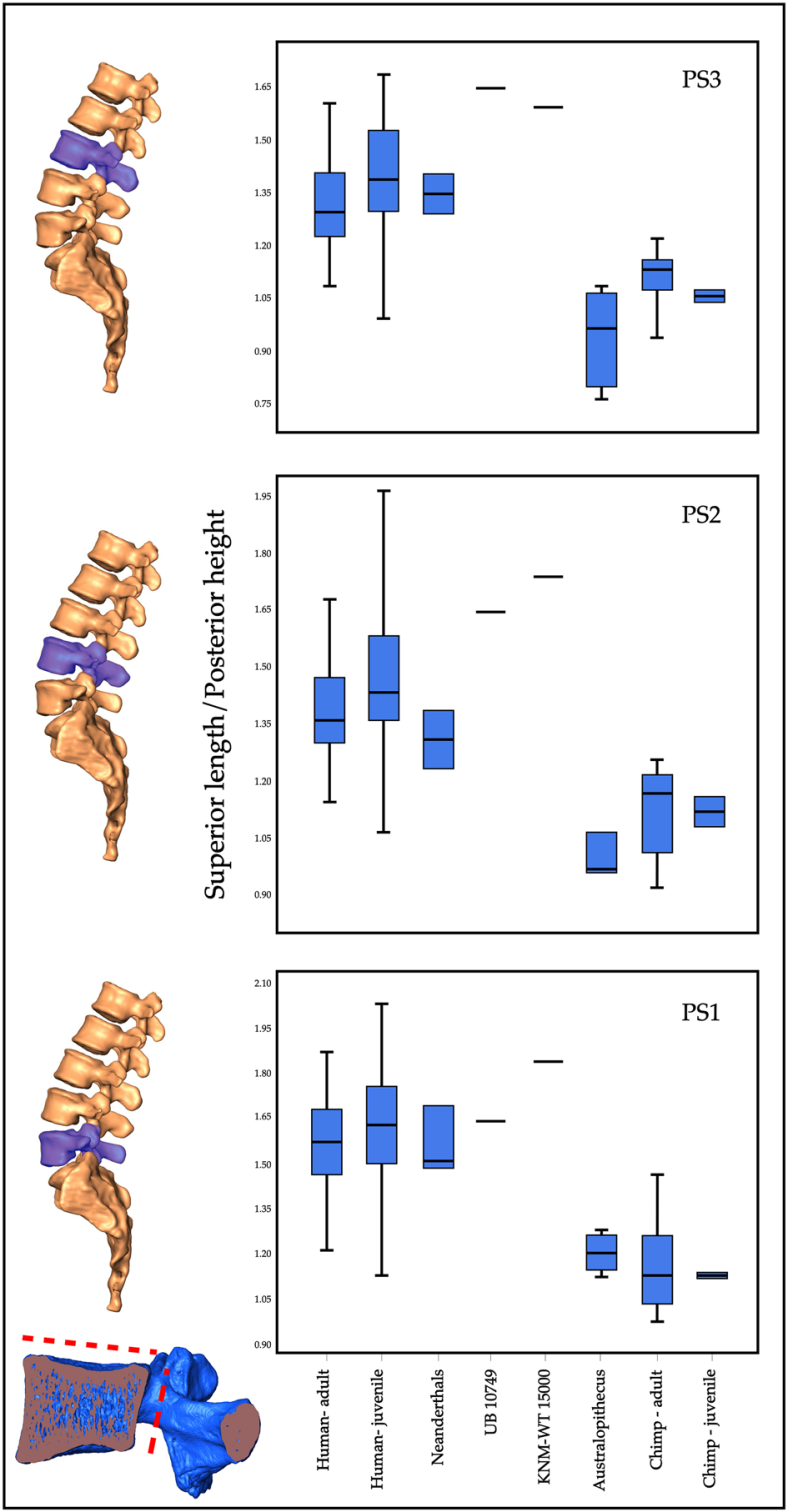
Comparison of UB 10749 to other hominoids. Vertebral body ratio (superior length to posterior height) of each of the lower 3 presacral vertebra in modern humans, neandertals, australopith, chimpanzees, KNM-WT 15000 and UB 10749. Note that UB 10749 is consistently falls within the range of *Homo*, and beyond the range of chimpanzees and australopith.

To narrow the taxonomic identification, we compared UB 10749 to a range of extant and extinct hominin species, and to *Pan* as an outgroup (Supplementary 2: Table 3). The analysis revealed that the best index to which best differentiates between lumbar vertebral bodies of *Homo* and *Pan* is 'superior length to posterior height' (Fig. 4; Supplementary 2: Table 4). This index also differentiates between *Homo* and *Australopithecus*^22^. Compared to the three presacral vertebrae (PS1–PS3) of hominins and *Pan*, UB 10749 falls within the range of *Homo* and outside the range of *Pan* or *Australopithecus*. It falls near the position of the vertebrae of KNM-WT-15000, an early Pleistocene sub adult specimen from east Africa. Therefore, we conclude that the vertebra at hand most probably belongs to an early Pleistocene *Homo*.

### Serial allocation of the vertebral body

**Figure 5:**
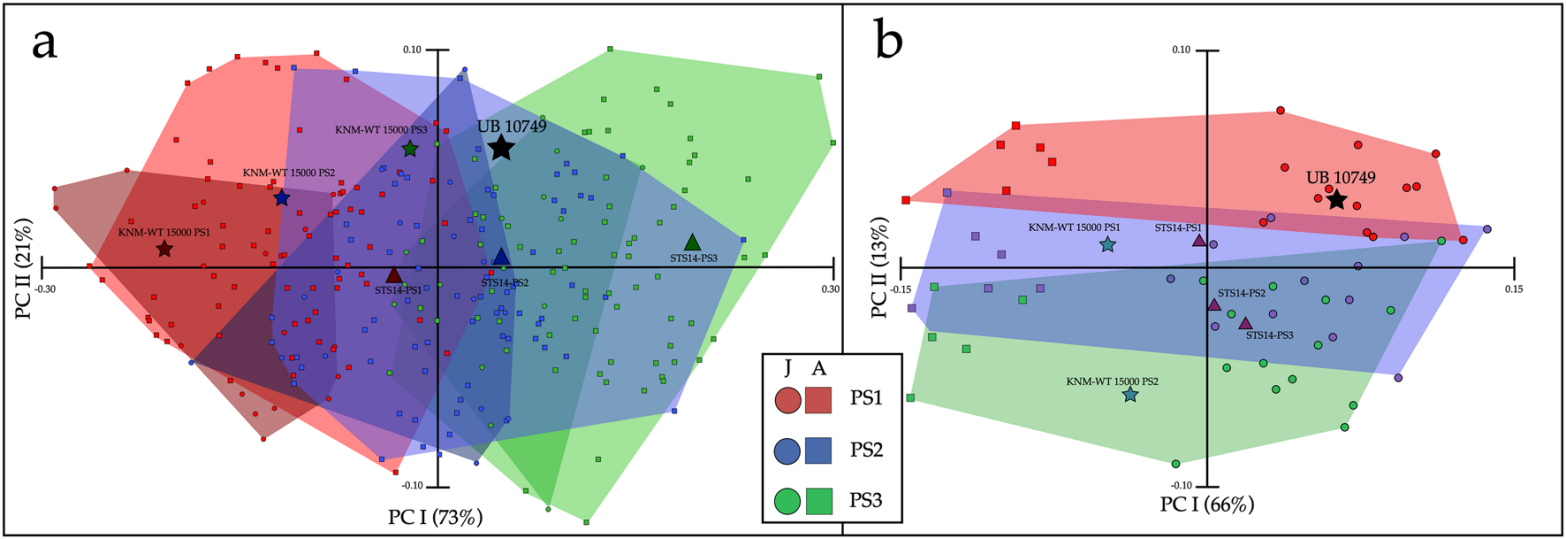
Serial allocation of UB 10749. (**a**) PCA of vertebral body ratios of modern humans, KNM-WT 15000, STS 14, and UB 10749 (see Supplementary Table 4). Note the overlap between adults and juvenile in each presacral vertebra. UB 10749 falls within the range of PS2–PS3. Note that KNM-WT 15000 and STS 14 follow the same pattern as modern humans. (**b**) PCA results for GM shape analysis. UB 10749 falls within the range of PS1, but with proximity to PS2. Note that KNM-WT 15000 and STS 14 follow the same pattern as modern humans. In both analysis: Circles denotes juvenile; Squares denotes adults. Red denotes PS1; Blue denotes PS2; Green denotes PS3.

It is well known, especially in Hominoidea, that there is a vast overlap in the shape of adjacent lumbar vertebral bodies^23^. We conducted three separate analyses to address this problem: (1) Vertebral wedging i.e., the ratio of posterior height/anterior height which significantly separates the vertebral segments PS1, PS2, and PS3 of modern humans (Supplementary 2: Fig. 1; Supplementary 2: Table 4), (2) A principal component analysis (PCA) of vertebral linear indices (Fig. 5a; Supplementary 2: Table 4), and (3) Geometric morphometrics (GM) shape analysis (Fig. 5b). Vertebral wedging sets UB 10749 as PS2. The vertebral linear indices PCA sets the UB 10749 as either PS2 or PS3, and the GM shape analysis sets the vertebra as either PS1 or PS2. Based on these results, we estimate that the serial allocation of UB 10749 is most likely PS2.

### Age at death

Age at death is estimated based on level of ossification, relative vertebral size, or vertebral shape. The lack of neural canal ossification in UB 10749 indicates an approximate age of 3–6-years-old compared to modern humans^24,25^ (Supplementary 2: Fig. 2), although it is important to note that several authors report high variability in pedicle ossification, up to 16-years-old^26,27^. The absence of vertebral endplate ossification also supports the young age of UB 10749, indicating that the vertebra belongs to an individual that had not reached puberty^28^.

**Figure 6:**
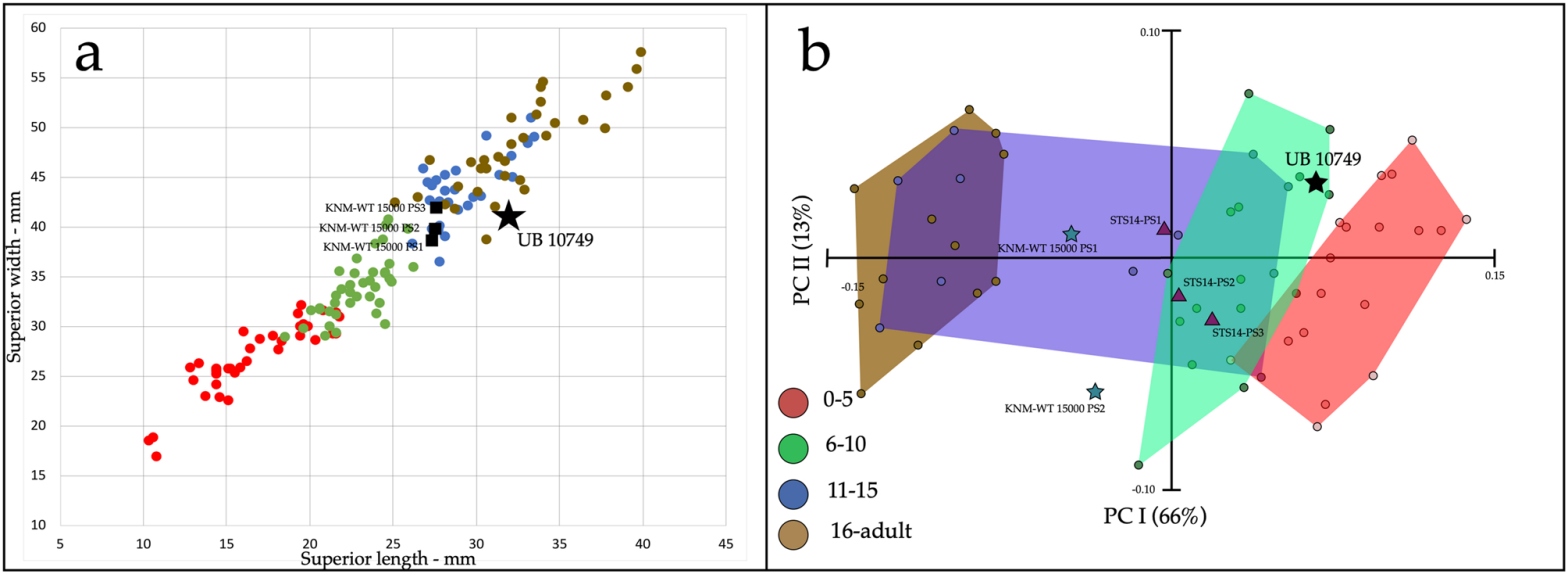
Age at death of UB 10749. (**a**) Vertebral body size (combined sample of PS1–PS3) of modern humans, KNM-WT 15000 and UB 10749 (see Supplementary 2: Table 5). UB 10749 falls within the range of 11–15 years or the lower end of adults. (**b**) PCA results for GM shape analysis of modern human, KNM-WT 15000, STS 14, and UB 10749 vertebral bodies. UB 10749 falls within the range of the 6–10 age group. In both analyses: Red, 0–5 years old; Green, 6–10 years old; Blue, 11–15 years old; Brown, 16-adults.

In contrast, based on its size alone, UB 10749 would be assigned an older age, probably between 11 and 15-year-old modern human (Fig. 6a: Supplementary 2: Table 5). However, vertebral size is highly variable with age, and we cannot rule out either a younger or older age. Finally, geometric morphometric principal component shape analysis suggests that UB 10749 falls within the range of 6–10-years-old modern humans (Fig. 6b). This is confirmed by a linear discriminant analysis which also places UB 10749 well within the 6–10 years old group (Supplementary 2: Fig. 4). Considering all the above information, we estimate that the age at death for UB 10749 is between 6 and 12-years-old.

### Height and weight estimation

Height (stature) and weight at death is estimated based on a range of equations and growth charts for modern humans (Supplementary 2: Tables 6–8). The estimated average height at death of UB 10749, points to a height of 155 cm. This height is comparable to a 13 years-old boy or a 12.5 years-old girl, based on CDC growth charts. A height of 155 cm is above the 95 percentile of 10 years old and above the 75 percentile for 12 years old modern humans^29^. As the age estimation for UB 10749 is 6–12 years, it seems that this individual was tall for its age.

Weight is estimated based on height or based on chronological age. Based on height, UB 10749 was 45–55 kg, while based on age, the weight of UB 10749 was 20–43 kg (Supplementary 2: Table 7). Since height is a stronger predictor for weight than age^30^, we estimate the weight at death at about 45–50 kg.

A single juvenile vertebral body is not a definitive predictor for adult height and weight. Even more so, the growth pattern of early Pleistocene hominins is unknown. Thus, to cautiously estimate the adult height and weight of UB 10749, calculations were based on several methods: modern American (CDC growth charts), modern Sudanese population^31^, and chimpanzees^32^.

Assuming UB 10749 was 6–12 years old, based on chimpanzees’ growth charts, it would have reached adult height of 155–192 cm and weighted 50–101 kg. Based on modern American and Sudanese growth charts, UB 10749 displays a range of a height between 168 and 247 cm and a weight between 62 and 173 kg (Supplementary 2: Table 8). The average height and weight predication for adult size of UB 10749 is 198 cm and 100 kg. Although we cannot rule out any of the estimations, based on its size at death and predicated adult size, UB 10749 was most likely a large-bodied hominin^33–35^.

### Taphonomy

Very thin fluviatile deposits are evident on the surface of the vertebra, despite being cleaned during the excavation. Aside from that, there is no apparent taphonomic alteration or post-depositional breakage.

### Pathology

The completeness of the vertebral body and its bilateral symmetry do not suggest pathological processes or developmental deformities that may have affected the vertebra, such as osteoarthritis, disc herniation, spondylosis, tuberculosis, brucellosis, or scoliosis^36^. However, in the absence of the vertebral arch, we cannot rule out anterior slippage of the vertebral body, i.e., spondylolisthesis or facet joint deformities. The discrepancy between the size of the vertebral body and the level of ossification is puzzling. The size of UB 10749 is equivalent to an 11–15-year-old modern human, and the level of ossification is equivalent to a 3–6-year-old modern human child. This discrepancy might result from several factors, including developmental or pathological conditions, such as: persistent notochondral canal; hypopituitarism; androgen deficiency; genetic mutation^24,37,38^ (see Supplementary 2 for discussion regarding possible pathology). While these conditions are rare in modern humans, they cannot be ruled out. Another possibility is that UB 10749 displays a different ossification pattern than observed in modern humans or great apes^25,39^.

## Discussion

### Paleobiology of UB 10749

The paucity of Eurasian skeletal remains from the early Pleistocene leaves a considerable gap in our understanding of hominin paleobiology. In this respect, the ‘Ubeidiya specimen is significant, providing important evidence regarding the diversity of hominins that dispersed out of Africa.

Its morphologic features indicate that UB 10749 is a lower lumbar vertebra of an early Pleistocene *Homo*. The dorsal wedging of the vertebral body, the widening of the inferior plate compared with the superior plate, the concavity of the inferior plate, and the ventrodorsally elongated vertebral body compared with vertebral height are all familiar lower lumbar human traits^13,20–22^.

The taxonomic affinity of early Pleistocene hominins is debated^40^, and suggested taxonomic nomenclature includes *H. rudolfensis*, *H. habilis*, *H. ergaster*, *H. erectus*, *H. georgicus*, and* H. antecessor*^41^. Within the early Pleistocene, two major taxon groups coexist. One, the small-bodied hominin, traditionally represented by *H. habilis* is characterized by small body, primitive limb proportions, robust skull and small cranial volume. This is opposed to the large-bodied hominin, represented by *H. erectus*, with human like limb proportions and larger cranial capacity^42,43^. Based on its size, the UB 10749 is too large to belong to the small-bodied hominins such as *H. habilis* sensu lato^33,43,44^. In this sense, UB 10749 is attributed to the large-bodied, early Pleistocene *Homo*. Since very little is known regarding their postcranial morphology, a more definitive taxonomical affinity is not possible.

The estimated height and weight at death for UB 10749 fit very well with those for KNM-WT 15000^45^. The latter is estimated to be an 8-year-old individual (based on dental development), with a height of 159 ± 7 cm (although see^46,47^ for different estimations) and weight of 49.2 ± 10 kg (based on the bi-iliac breadth^34^), compared to 155 cm and 45–50 kg for UB 10749. KNM-WT 15000 is a tall individual with a level of ossification advanced for its chronological age^48^. In contrast, UB 10749 displays different ontogenetic patterns with delayed ossification and increased size, implying that UB 10749 would have grown larger than KNM-WT 15000.

Comparing KNM-WT 15000 and UB 10749 to the Dmanisi individual D2700/D2735, the latter is smaller, at 41 kg and 153.1 cm. However, D2700/D2735 had almost reached its adult size, as adult specimens from Dmanisi have yielded overall estimates between 145–166 cm and 40–50 kg^49^.

The evidence presented here demonstrates that UB 10749 represents a hominin with shared affinities to East African large-bodied hominins such as KNM WT 15000, KNM-ER 736, and KNM-ER 1808, MK3(IB7594)^33,44,50^ and is therefore the earliest definitive evidence of a large-bodied hominin in the Levantine corridor.

### Paleoecological implications

Establishing the tempo and mode of hominin dispersal from Africa is critical in paleoanthropology^51–53^, with considerable research devoted to understanding the push and pull forces influencing hominin dispersal. While it has been assumed that there were many dispersals events^3,54^, the dispersals are often discussed in terms of filling a single ecological niche for early *Homo* sensu latu. Moreover, hypotheses concerning intrinsic or extrinsic forces that may have led or supported these dispersals are often tested across sites dating from 1.9 to 0.8 Mya but analyzed as a single dispersal event. However, other researchers suggest that the difference in lithic assemblages between Dmanisi (Oldowan) and ‘Ubeidiya (Acheulean) reflect separate dispersal events^55^.

Our conclusion that UB 10749 is a large-bodied Levantine hominin, supports the occurrence of several Pleistocene dispersals that were not only separated in time but also in ecology. Dmanisi is reconstructed as open grassland, with arid conditions compared to today^56,57^. In contrast, the younger site of ‘Ubeidiya is reconstructed as warmer but more humid than Dmanisi, with closed woodland forests^58^. It follows that each of the hominin populations associated with the dispersals may have exhibited unique ecological and behavioral adaptations. Most importantly, our interpretation that the large-bodied hominin from ‘Ubeidiya and the small-bodied hominin from Dmanisi were not from the same population explains the difficulty thus far in identifying a single early Pleistocene *Homo* niche^59,60^. Chronologically, Dmanisi predates ‘Ubeidiya by several hundred thousand years (200–500kya, see supplementary 1). It is possible that there was a single dispersal event, followed by in-situ local evolution which led to different hominin morphology in to two regions. This explanation is unlikely as we find small and large bodied hominins coexisting within Africa during the early Pleistocene for nearly 500kya^61^. Therefore, the more parsimonious explanation is two distinct “Out of Africa” dispersal events.

Going forward, when analyzing the early Pleistocene “Out of Africa” event, we should be cognizant that more than a single hominin population may have dispersed from Africa, each time with their own diverse biological and cultural adaptations.

## Materials and methods

### Materials

UB 10749 was found in 1966, from Layer II-23. This layer was excavated from 1960 to 1966 by Stekelis, and again in 1970 by Bar Yosef^62–65^ (Supplementary 1). UB 10749 was identified during a reanalysis of faunal remains, which are housed at the National Natural History Collections, the Hebrew University of Jerusalem. The fossilized vertebra has a red-brown color (Fig. 2), consistent with the soil color of layer II-23^65^.

### Qualitative observation

UB 10749 was observed using three modalities: Macroscopic description, micro-CT scan (Triumph® II, triFoil Imaging), and a digital microscope (Dino-lite edge digital microscope). A comparative sample of large mammals was studied at the Steinhardt Museum for Natural History, Tel Aviv University, and the National Natural History Collections, Hebrew University of Jerusalem.

### Linear measurements

Our comparative sample included adult and sub-adult of modern humans (103; 73 adults, 30 sub adults), extinct hominins (13; 10 adults, three subadults), and chimpanzees (14; 12 adults, two subadults) (Supplementary 2: Table 3). We conducted linear measurements on osseous material using Mitotuyu caliper and CT scans from the Sheba Medical Center imaging database (Helsinki committee approval number: 8266 10 SMC) using Horos software (Horosproject.org). For each vertebra, we conducted six Linear measurements: (1) anterior height, (2) posterior height, (3) superior length, (4) superior width, (5) inferior length, (6) inferior width (Supplementary 2: Table 2). Single linear measurements may not indicate taxonomy, age, and seriality due to significant variation in size. Therefore, we also calculated four indexes for each vertebra: (1) anterior height/posterior height, (2) superior length/superior width, (3) inferior length/inferior width, and (4) superior length/posterior height (Supplementary 2: Table 4). For age estimation, superior length and superior width were measured on CT scans of additional 50 individuals with known age from the Sheba Medical Center Imaging database (Supplementary 2: Table 5).

### Geometric morphometrics

11 key landmarks were placed by one of the authors (A.B.) on the vertebral bodies using dHAL Viewbox (ver. 4.0.1.7, http://www.dhal.com/index.htm). On the superior and inferior plates: most anterior point; most posterior point; and two lateral points on the outmost edges of the vertebra. Three additional points were placed on the anterior and lateral walls and the midpoint between the two plates. Additionally, eighty sliding-semilandmarks were automatically placed on the vertebral body (Supplementary 2: Fig. 3). For GM analysis, we studied vertebral bodies of 20 humans, ranging from 3.5 to 27 years old (PS1–PS3); reconstructed vertebral bodies of STS-14 (PS1–PS3) (Supplementary 2: Fig. 5); KNM-WT 15000 (PS1–PS2) and UB 10749. In EVAN Toolbox (https://www.evan-society.org/), specimens were superimposed using the Procrustes method. Principal component analysis (PCA) was conducted on the dataset to detect shape-related changes of seriality and age. The first PC (66%) correlates to age-related changes and the second PC (13%) Correlates to vertebral seriality (Figs. 5 and 6). Linear discriminant analysis was performed on the superimposed data (Supplementary 2: Fig. 4), to detect modern human age groups, and the position of UB 10749 in relation to these groups.

## Supplementary Information

Supplementary Information 1.
