# Supplementary material for: The earliest Pleistocene record of a large-bodied hominin from the Levant supports two out-of-Africa dispersal events
Source: Sci Rep. 2022 Feb 2;12:1721. doi: 10.1038/s41598-022-05712-y (PMC8810791; doi:10.1038/s41598-022-05712-y)
Supplement: Supplementary file 1 — Supplementary Information 1. [file 41598_2022_5712_MOESM1_ESM.docx]

### Supplementary material

The supplementary includes the following:

1. **The site of 'Ubeidiya:**

**Stratigraphy**

**Layer II-23**

Supplementary 1, Table 1. The lithic assemblage of Layer II-23.

Supplementary 1, Fig. 1. Artifacts made on basalt from layer II-23.

Supplementary 1, Fig. 2. Artifacts made on basalt, hard limestone, and flint from layer II-23.

Supplementary 1, Fig. 3. Artifacts made on flint from layer II-23.

**Dating**

**Fauna and biochronological considerations**

Supplementary 1, Table 2. The Fauna from Layer II 23 from 'Ubeidiya.

**Human behavior**

1. **Extended data on UB 10749:**

**Vertebral ontogeny and pathology.**

**Tables (1-8):**

Supplementary 2, Table 1. Major Early Pleistocene archeological sites with osseous human remains.

Supplementary 2, Table 2. Linear measurements for modern humans, chimpanzees, extinct hominins and UB 10749 for three spinal vertebrae PS1, PS2, and PS3.

Supplementary 2, Table 3. List of specimens studied in the current research.

Supplementary 2, Table 4. Ratios/Indexes of vertebral dimensions for modern humans, chimpanzees, extinct hominins and UB 10749 for three spinal vertebrae: PS1, PS2, and PS3.

Supplementary 2, Table 5. Vertebral body size (PS1-PS3) of modern human children and adults with known age. Data was acquired from CT scans.

Supplementary 2, Table 6. Height estimation at death for UB 10749.

Supplementary 2, Table 7. Weight estimation at death for UB 10749, assuming an age of death of 6-12 years, and a height of 155 cm.

Supplementary 2, Table 8. Predicted adult size (height and weight) for UB 10749 based on growth charts of chimpanzees and modern humans.

**Figures:**

Supplementary 2, Fig. 1: Vertebral body wedging (posterior/anterior height ratio) of UB 10749 and adult modern humans (for three lumbar vertebrae PS1-PS3). Note that the ratio of UB 10749 is most similar to that of PS2.

Supplementary 2, Fig. 2: Pedicle ossification level across ages.

Supplementary 2, Fig. 3: Anterolateral view of the UB 10749 showing the location of the landmarks and semi landmarks used in the study.

Supplementary 2, Fig. 4: Reconstruction of *Australopithecus africanus* STS 14 (PS1-3).

1. **References**

### **The site of ‘Ubeidiya**

The Pleistocene deposits of ‘Ubeidiya were first discovered in 1959, and a series of excavations were conducted from 1960 -1974, 1989- 1994, and 1997- 1999. Due to the anticline structure and the tilted stratigraphy, the only technique to expose the various layers or beds was employing heavy machinery. Several geological trenches (numbered I-V, K and KA) were excavated with a total length of ~1100 meters^1-4^. The archaeological excavations at ‘Ubeidiya uncovered numerous layers with artifacts^4^. Of the 65 observed lithic- and bone-bearing layers, 15 major archaeological horizons were excavated over sufficiently large exposures to provide a relatively comprehensive lithic and faunal assemblage^4^.

**Stratigraphy**

The observed sequence was subdivided into four cycles based on their facies: two limnic (Li and Lu) and two terrestrial cycles (Fi and Fu)^1,2^:

1. The Li-cycle, characterized by clays, silts, and limestone, terminates with laminated silts rich with freshwater mollusks and fish remains. One layer (III-12) contained mammalian bones, crocodiles, as well as artifacts, and provided the only pollen spectrum indicating a Mediterranean forest cover on the surrounding hills^3^.
2. The Fi-cycle is composed of clays and conglomerates, mainly beach deposits. Most archaeological finds and faunal remains were obtained from this member, beginning with layers II-21 through III-64^4^.  UB 10749 was found in layer II-23 attributed to the Fi-cycle.
3. The Lu-cycle is the upper limnic member. It consists of two parts: the lower part comprises clays and chalks, and the upper part is a white-greyish-yellow silty series. Only a few artifacts were encountered in this unit.
4. The Fu-cycle consists mainly of conglomerates with some large basalt boulders. No artifacts or mollusks were found in this member. Presumably, it represents the regression of Lake ‘Ubeidiya due to either regional drying or the impact of local tectonics. This member is capped by Middle Pleistocene rocks and sediments emplaced by a landslide.

**Layer II-23**

The layer was initially excavated in Area A during the 1960-1966 campaign by Stekelis^5^. The field methodology at that time followed excavation in a 5X5 m^2^ grid which was set next to the trenches excavated by heavy machinery. Plotting of artifacts was done according to squares and geological units but without reference to the strikes of the layers at the site^4^. Layer II-23 was re-excavated in 1970 by Bar-Yosef and Tchernov following a different field method which included 1X1 m^2^ grid which was set on the geological units according to their strike. The total exposure of layer II-23 corresponds to 105 m^2^.

The sediments of layer II-23 are described as a rusty brown siltic clay ^1,2,4,5^. The finds within this layer consist of vertebrate fauna and lithic artifacts. The lithic assemblage from layer II-23 is briefly reported in Bar-Yosef and Goren Inbar^4^. A reexamination of the lithic assemblage of layer II-23 was carried in summer 2021. A total of 34 artifacts were counted - 30 from the 1960-1966 excavations and four from the 1970 excavation (Supplementary 1, Table 1).

The lithic assemblage is composed of three raw material types – flint (n=29), basalt (n=4) and hard limestone (n=1) (Supplementary 1, Table 1). The basalt items include a bifacial preform, a large flake, a chopper and a trihedral (Supplementary 1, Figs. 1:1-2; 2:1). The limestone artifact is a broken hammerstone/pounder (Supplementary 1, Fig. 2:2). The flint artifacts are in very fresh condition and consist mainly debitage flakes and core trimming elements but also bear cores and choppers (Supplementary 1, Figs. 2:3; 3:1-6). The composition of the artifacts corresponds to assemblages from other strata in 'Ubeidiya and confirm to the definition of early Acheulian.

**Supplementary 1, Table 1. The lithic assemblage of Layer II-23**

| **#** | **context** | **type** | **raw material** | **note** | **preservation** |
| --- | --- | --- | --- | --- | --- |
| 1 | U/63/22 | Two side chopper | flint |  | fresh |
| 2 | U/63/147 | Two side chopper | flint | flake core? | fresh |
| 3 | U/62/267 | Two side chopper | flint | flake core? | patinated |
| 4 | U/60/7 | Flake | basalt |  |  |
| 5 | U/63/99 | Trihedral | basalt | oxidized |  |
| 6 | U/61/3 | Preform (handaxe) | basalt |  |  |
| 7 |  | Pounder/chopper | basalt | oxidized |  |
| 8 | U/63/236 | Flake core | flint |  | fresh |
| 9 | U/63/149 | Hammerstone | limestone | broken |  |
| 10 | U/63/325 | Notch | flint | made on flake | fresh |
| 11 | U/60/10/1 | Cortical blade | flint | backed knive | fresh |
| 12 | U/63/139 | Fragment | flint |  | fresh |
| 13 | U/63/36 | Core trimming element | flint | made on flake | fresh |
| 14 | U/70 | Modified | flint | mini pebble |  |
| 15 | U/60/38 | Preform (handaxe) | flint |  | fresh |
| 16 | U/60/28 | Flake | flint |  | fresh |
| 17 | U/63/11 | Flake | flint |  | fresh |
| 18 | U/60/37 | Core | flint | exhausted | fresh |
| 19 | U/63/30 | Primary flake | flint |  | fresh |
| 20 | U/70 | Flake | flint |  | rolled |
| 21 | U/63/35 | Flake | flint | chacedony | fresh |
| 22 | U/63/287 | Flake | flint |  |  |
| 23 | U/63/37 | Flake | flint |  |  |
| 24 | U/65/8 | Scraper | flint | zebra flint, broken | |
| 25 | U/63/291 | Flake | flint |  |  |
| 26 | U/60/10 | Core trimming element | flint | made on flake |  |
| 27 | U/70 | Flake | flint | broken |  |
| 28 | U/63/323 | Flake | flint | hinge removal flake | fresh |
| 29 | U/63/148 | Flake | flint |  | fresh |
| 30 | U/63/38 | Core trimming element | flint | made on flake | fresh |
| 31 | U/62/10 | Core trimming element | flint | made on blade | fresh |
| 32 | U/70 | Fragment | flint |  | fresh |
| 33 |  | Flake | flint |  | fresh |
| 34 |  | Fragment | flint | pebble fragment |  |

**Supplementary 1, Fig. 1.** Artifacts made on basalt from layer II-23. 1. Flake; 2. Handaxe preform. Photo credit: Dafna Gazit, Israel Antiquities Authority.

**
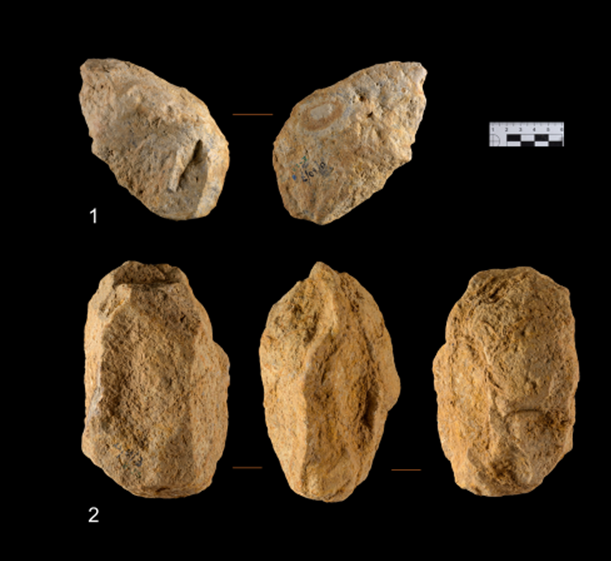
**

**Supplementary 1, Fig. 2.** Artifacts made on basalt, hard limestone, and flint from layer II-23. 1 trihedral; 2 broken hammerstones; 3 chopping tools. Photo credit: Dafna Gazit, Israel Antiquities Authority.

**
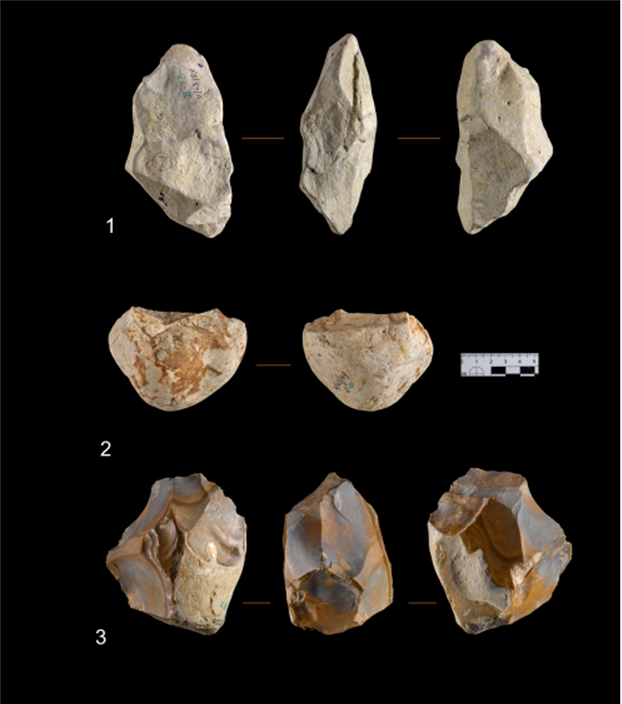
**

**Supplementary 1, Fig. 3.** Artifacts made on flint from layer II-23. 1 scraper (broken); 2 cortical blades; 3-5 flakes; 6 core trimming elements. Photo credit: Dafna Gazit, Israel Antiquities Authority.

**
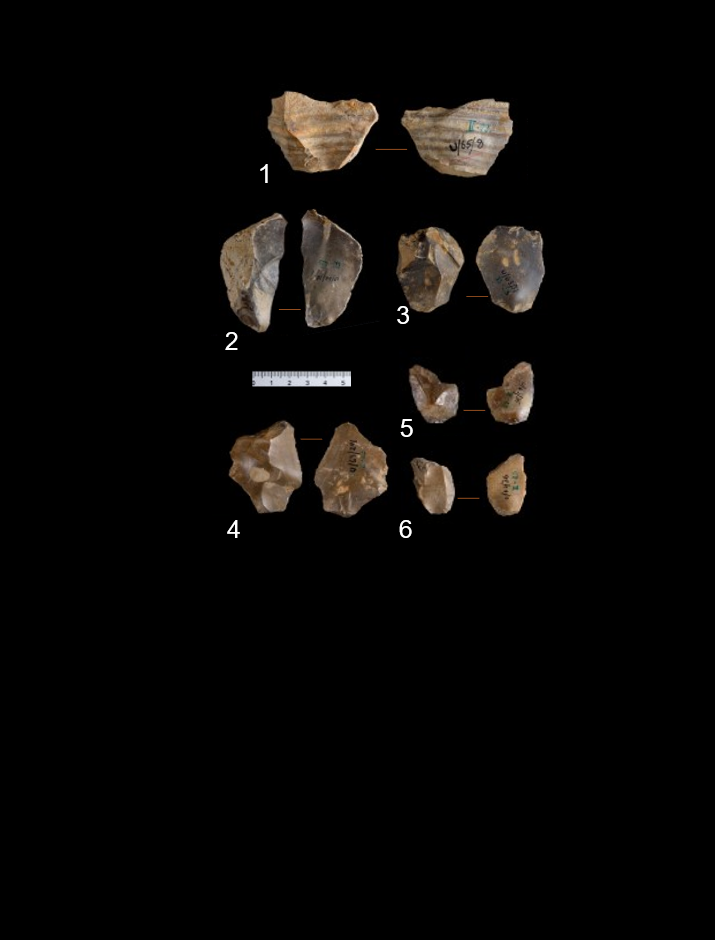
**

**Dating**

The 'Ubeidiya Formation's relative chronology is based on its relative position within the Kinnarot Basin in the Dead Sea Fault and is considered as follows from oldest to youngest:

a. The cover basalt has been dated using Ar/Ar to 3.99 Ma^6^.

b. The lacustrine and fluvial sediments of the Erq el-Ahmar Formation indicate the remains of an earlier freshwater lake that predates Lake 'Ubeidiya^7^. These sediments were dated by paleomagnetic records and cosmogenic nucleotide isotopes to 5.3-3.5 Ma^6,8^. Furthermore, the upper Erq el Ahmar Formation^9^ was dated to the Late Pliocene age due to the presence

of the fossil*hydrobia acuta*and *Dreissena chantrei* snf eight extinct species not found in 'Ubeidiya ^9-11^.

c. The 'Ubeidiya Formation was deposited as a deltaic formation following a tectonic movement that contorted the formation. Paleomagnetic analysis of the 'Ubeidiya Formation indicated a reversed polarity suggesting that it predates the Brunhes - Matuyama reversal^12^. Two short, normal paleomagnetic episodes have been found in the Fi member layers II-33 and II-23-24. Layer II-33 has been assigned to the Cobb Mt. (1.215 - 1.190 Ma), and the putative normal episode in layer II 23-24 has been assigned to the Gilsa (1.575-1.567 Ma)^13,14^. This correlation fits well with an ESR date of ca. 1.2 Ma for layer I-26, which overlays layer II-33^15^. These results correlate well with the long-range biochronological results.

d. The post cover Yarmuk Basalt, bearing a normal polarity, does not directly overlie the 'Ubeidiya Formation but is considered to postdate the latter based on geologic correlations^7^ and has been dated to 0.85 Ma^6^.

#### Fauna and biochronological considerations

A total of 679 large mammal fragments were recovered from layer II-23 and identified by several authors^11,16-18^. Non mammalian fauna included significant quantities of birds, reptiles, amphibia, and fish (Supplementary 1, Table 2).

**Supplementary 1, Table 2**. The Fauna from Layer II 23 from 'Ubeidiya.

| Order | Suborder | Family | Genus and species | Number of identified specimens |
| --- | --- | --- | --- | --- |
|  |  |  |  |  |
| Carnivora family indet. |  |  |  | 49 |
|  |  | Canidae |  |  |
|  |  |  | *Lycaon lycaonoides* | 4 |
|  |  |  | *Canis mosbachensis* | 33 |
|  |  |  | *Vulpes* cf. *praeglacialis* | 8 |
|  |  | Mustelidae |  |  |
|  |  |  | *Lutra simplicidens* | 6 |
|  |  |  | *Vormela peregusna* | 3 |
|  |  | Felidae |  |  |
|  |  |  | *Panthera gombaszoegensis* | 1 |
|  |  |  | *Megantereon* cf. *whitei* | 1 |
|  |  |  | *Lynx sp.* | 8 |
|  |  |  | *Felis* cf. *chaus* | 1 |
|  |  | Herpestidae |  |  |
|  |  |  | *Herpestes* sp. | 2 |
|  |  | Ursidae |  |  |
|  |  |  | *Ursus etruscus* | 2 |
| Artiodactyla family indet. |  |  |  | 12 |
|  | Suiformes |  |  |  |
|  |  | Suidae |  |  |
|  |  |  | *Sus strozzi* | 3 |
|  |  |  | *Kolpochoreus oldowaynesis* | 1 |
|  |  | Hipppotamidae |  |  |
|  |  |  | *Hippopotamus gorgops* | 2 |
|  |  |  | *Hippopotamus* cf. *behemoth* | 166 |
|  | Ruminantia indet. |  |  | 91 |
|  |  | Cervidae gen and sp. indet. |  | 27 |
|  |  |  | *Praemegaceros verticornis* | 38 |
|  |  |  | *Pseudodama nestii* | 127 |
|  |  |  | *Capreolus* sp. | 10 |
|  |  | Bovidae |  |  |
|  |  |  | *Pelorovis oldowayensis* | 3 |
|  |  |  | *Bovini* sp. | 7 |
|  |  |  | *Antilopini* indet. | 5 |
|  |  |  | *Gazella* sp. | 8 |
| Perissodactyla |  |  |  |  |
|  |  | Rhinocertidae |  |  |
|  |  |  | *Stephanorhinus*  *etruscus etruscus* | 10 |
|  |  | Equidae |  |  |
|  |  |  | *Equus tabeti* | 73 |
|  |  |  | *Equus* sp. indet. | 20 |
| Proboscidea |  |  |  |  |
|  |  | Elephantidae |  |  |
|  |  |  | Mammuthus meridionalis | 5 |
| Primates |  |  |  |  |
|  |  | Cercopithecoidea |  |  |
|  |  |  | *Macaca sylvanus* | 2 |

Long-range biochronological correlation on the fauna of ‘Ubeidiya as whole suggests a late Villafranchian European mammalian age (ca. 1.8 - 1.1 Ma). Classic late Villafranchian species include Etruscan bear (*Ursus* *etruscus*), dirk-toothed felid (*Megantereon* cf. *whitei*), Meridian mammoth (*Mammuthus meridionalis*) as well as Etruscan rhino (Step*hanorhinus etruscus etruscus*). More specifically, the ‘Ubeidiya fauna corresponds to Mammal Neogene Quaternary (MNQ) biozones MNQ 16 through MNQ 20^19^. Within this long period, the precise position of ‘Ubeidiya is challenging to ascertain. No species indicative of MNQ zone 18 (ca. 1.9 - 1.4 Ma), e.g., *Croizetoceros ramosus* minor, *Cervus philisi philisi*, *Eucaldoceros senezensis* have been found in ‘Ubeidiya. Similarly, no specimens of Step*hanorhinus etruscus brachycephalus*indicative of MNQ zone 20 (ca. 1.0 - 0.6 Ma) have been found at ‘Ubeidiya. Comparison with the Italian faunal units suggest the most significant similarities of the ‘Ubeidiya faunal assemblage are with the Farenta faunal unit sites (the site of Selvella and Pieterfitta, Italy), which are dated to 1.6-1.2 Ma^20^. Although ‘Ubeidiya shares some species (e.g., *Lycaon* *lycaonoides* = *Xenocyaon falconeri*) with the following faunal unit of Pirro Nord (ca. 1.2 - 1.0 Ma), Pirro Nord should be considered younger than ‘Ubeidiya based on the replacement of *Ursus etruscus* with advanced arctoid bears^20^. Thus, that the age of `Ubeidiya should be best confined between ca. 1.6 and 1.2 Ma^21^.

#### Human behavior

While few cutmarks were uncovered on large mammals from layer II-23^22^, the low proportion of cut-marks and the high proportion of gnaw-marks on mid-shaft fragments are more consistent with scavenging by large carnivores, such as large felids, rather than hunting by early hominin. The absence of evidence for bone marrow processes may be attributed to a reverse utility and processing off-site^17^. This model does not negate the possibility that some individual specimens were acquired by active hunting, but it does suggest that this was not the common practice by the early hominins at ‘Ubeidiya. Furthermore, taphonomic analysis of the large mammals suggests that weathering and fluvial transport were not primary factors in their accumulation. The high abundance of specimens assigned to weathering stages 1 and 2 suggests that most specimens were buried quickly^17^. The highly fragmented state of the cranial elements usually not consumed by carnivores point to a high degree of post-depositional mechanical fragmentation resulting from trampling and soil compaction.

### **Extended data on UB 10749**

### **Vertebral ontogeny and pathology**

The discrepancy between the size and shape of the vertebral body compared to the level of ossification is puzzling. While the size of UB 10749 is equivalent to 11–15-year-old modern human, the absence of pedicle ossification points to a 3–6-year-old modern human child. This discrepancy together with the presence of notochondral canal, might result from several factors, including developmental pathological conditions. Mellado et al.^23^ report that congenital absence and hypoplasia of vertebral pedicles is more frequent in the lumbar spine compare with thoracic or cervical spine. Rajwani et al.^24^ report high variability on the exact age of pedicle closure (neurocentral synchondrosis, NCS). Most of the research shows that the NCS is actively open at a very early age and closes during childhood (3-6 years of age), but there is evidence of NCS closure up to the age of 16 years^25^. Delayed pedicle ossification is also attributed to congenital factors, hypopituitarism, or androgen deficiency^25-27^.

The two pits at the superior and inferior plates and the non-ossified column that connects them is known in the orthopedic literature as persistent notochondral canal. This is a rare, usually asymptomatic malformation, seen in embryos but can be occasionally found in adult humans as well^28,29^. Taylor^30^ studied the notochondral canal in embryos and children up to ten years old. He found non ossification of the notochondral canal at about 7% of the children. He argued that this finding is due to a transient delay in ossification in children and that it will regress with age. Postma et al^31^. have shown that a persistent notochordal canal and abnormal ossification of the vertebral bodies associates with mutations in the T (brachyury) gene. Furthermore, delayed ossification and the presence of notochondral canal, relative to size and shape, may have been caused by other etiologies such as poor nutrition, genetic mutations, and endocrinological pathologies. However, most of the pathologies associated with delayed spinal ossification are rare in humans and to the best our knowledge has not been described in the fossil record.

Malnutrition and chronic diseases are another etiology for delayed skeletal maturity. Lewis et al.^26^ report a delay in skeletal maturity and epiphyseal closure in Malawian children due to poor nutrition and chronic diseases such as malaria and diarrhea. Yet, Martorell et al. ^32^ showed that while malnutrition causes delay in height, it has a smaller effect on ossification centers. The large vertebral body of UB 10749, compare to its level of ossification displays the opposite pattern. It indicates that the discrepancy between the size and the level of ossification is not due to poor nutrition.

If UB 10749 is a non-pathological vertebra its morphology may indicate a different growth pattern that existed in this clade of hominins. One possibility is that this is a young individual that grew up rapidly and based on its level of ossification would have grown to be a very large individual at maturity. The second possibility is that this is a sub adult specimen that nearly reached its maximal height but had a different ossification pattern from that of extant hominoids, as both humans and great apes display early ossification of their pedicles^33^. Interestingly, Rosas et al.^34^ and Cunningham et al.^35^ had previously suggested a delayed vertebral ossification of Neandertals and early African *Homo*, indicating variably in ossification rates in hominins.

#### Supplementary 2, Tables.

#### Supplementary 2, Table 1. Major Early Pleistocene archeological sites with osseous human remains.

| Name | Location | Osseous findings | Geological age | Suggested taxonomy | Vertebral remains |
| --- | --- | --- | --- | --- | --- |
| Dmanisi^36-40^ | Georgia | Skulls and post crania of at least five individuals | 1·78–1·85 Ma | *H. erectus/H. georgicus* | Isolated vertebra |
| Gona^41^ | Afar, Ethiopia | Pelvis, partial vertebra | 1.8 Ma | *H. erectus* | Neural arch of last lumbar vertebra, partial sacrum |
| Modjokerto and Sangiran^42-45^ | Java | Mostly cranial remains | 1.8–1.6 Ma | *H. erectus* | - |
| Venta Micena*^46-48^ | Orce, Granada, Spain | Long bones | 1.65 Ma | ? | - |
| Koobi Fora^49^ | Kenya | Skull remains | 1.4 - 1.9 Ma | *H. erectus* sensu lato | - |
| West Turkana, Nariokotome^50^ | Kenya | KNM – WT 15000, Nearly complete skeleton | 1.53 Ma | H. erectus | Nearly complete thoracic and lumbar spine, partial sacrum |
| 'Ubeidiya^4^ | Israel | Isolated teeth, skull fragment, lumbar vertebral body | 1.2-1.6 Ma | early *Homo* | Lower lumbar vertebral body |
| Barranco León^51^ | Orce, Granada, Spain | first deciduous molar | 1.4 Ma | early *Homo* | - |
| Sima de Elefante^52-54^ | Burgos, Spain | Mandible (ATE9-1), a manual phalanx, a small fragment of a femur | 1.0 - 1.3 Ma | *H. antecessor* | - |
| Uadi Aalad and  Mulhuli-Amo (Buia)^55,56^ | Danakil, Eritrea | Adult cranium, three pelvic remains, two isolated permanent incisors. | 1.0 Ma | *Homo sp.* | - |
| Cueva Victoria*^57,58^ | Murcia, Spain | Phalanx | 0.85Ma | ? | - |
| Gran Dolina^59,60^ | Burgos, Spain | Skull and post cranial remains | 0.78 Ma | *H. antecessor* | Isolated vertebra |

*Questionable attribution to *Homo* species

**Supplementary 2, Table 2.** Linear measurements for modern humans, Chimpanzees, extinct hominins and UB 10749 for three spinal vertebrae PS1, PS2, and PS3.

|  | Measurement | Superior length  X ± *SD* (mm) | Superior width  X ± *SD* (mm) | Inferior length  X ± *SD* (mm) | Inferior width  X ± *SD* (mm) | Anterior height  X ± *SD* (mm) | posterior height  X ± *SD* (mm) |
| --- | --- | --- | --- | --- | --- | --- | --- |
| Specimens | | 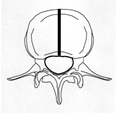 | 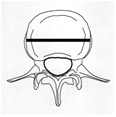 |  |  | 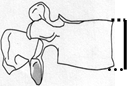 | 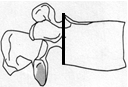 |
| UB 10749 | | 32.0 | 41.1 | 34.0 | 47.5 | 21.9 | 19.3 |
| PS1 | | | | | | | |
| Modern human | Adult  N = 73 | 33.6 ± 2.9 | 49.7 ± 4.1 | 32.5 ± 2.9 | 49.7 ± 4.7 | 26.4 ± 1.7 | 21.4 ± 1.6 |
|  | Juvenile  N = 30 | 26.2 ± 4.7 | 40.8 ± 7.8 | 23.4 ± 3.9 | 37.7 ± 5.3 | 20.4 ± 5.5 | 16.7 ± 4.9 |
| Pan | Adult  N =12 | 27.2 ± 5.9 | 39.3 ± 8.1 | 25.9 ± 5.4 | 39.1 ± 9.1 | 23.7 ± 4.8 | 23.8 ± 4.7 |
|  | Juvenile  N = 1 | 17.2 | 25.4 | 15.4 | 24.4 | 14.2 | 15.3 |
|  | Infant  N = 1 | 12.1 | 19.8 | 11.7 | 19.7 | 11.1 | 10.6 |
| Neandertal | Kebara 2 | 35 | 51.9 | 33.2 | 50.1 | 29.1 | 23.4 |
|  | Shanidar 3 | 38.9 | 55.6 | 37.95 | 55.11 | 29.49 | 22.9 |
|  | La Chapelle aux Saints | 34.9 | 59.0 | 32.9 | 58.0 | 29.8 | 24.5 |
| H. erectus | Nariokotome | 27.6 | 41.9 | 27 | 41.8 | 19.7 | 14.9 |
| *Australopithecus* | Sts 14 | 19.0 | 27.7 | 16.3 | 25.0 | 18.8 | 16.8 |
|  | Stw 431 | 23.1 | 37 | - | - | 22.2 | 19.8 |
|  | SK 853/3981 | 23.7 | 39.7 | - | - | 21.3 | 19 |
|  | Sediba MH2 | 21.1 | 31.1 | 19.4 | 27.9 | 19.6 | 16.4 |
|  | StW 600 | 23.8 | 40.2 | 22.9 | 34 | 22.3 | 19.7 |
| PS2 | | | | | | | |
| Modern human | Adult  N = 73 | 33.3 ± 2.7 | 47.9 ± 4.3 | 33.3 ± 3.0 | 51.0 ± 4.4 | 26.0 ± 1.6 | 24.0 ± 1.5 |
|  | Juvenile  N = 30 | 26.0 ± 4.5 | 37.9 ± 6.6 | 24.2 ± 3.8 | 37.1 ± 5.2 | 19.8 ± 5.2 | 18.5 ± 5.9 |
| Pan | Adult  N =12 | 27.7 ± 6.8 | 38.3 ± 7.9 | 28.2 ± 6.4 | 39.9 ± 8.8 | 23.4 ± 4.4 | 24.6 ± 4.3 |
|  | Juvenile  N = 1 | 16.1 | 21.9 | 15.6 | 25 | 14.1 | 14 |
|  | Infant  N = 1 | 12 | 19.4 | 12.4 | 21 | 11.8 | 11.2 |
| Neandertal | Kebara 2 | 38 | 51.1 | 37.5 | 51.5 | 26.2 | 27.6 |
|  | Shanidar 3 | 35.4 | 53.2 | 36.1 | 54.1 | 29.2 | 29.0 |
|  | La Chapelle aux Saints | 39.5 | 57 | 38.6 | 56.4 | 26.5 | 26.1 |
| H. erectus | Nariokotome | 27.5 | 39.8 | 28.5 | 41.2 | 19.2 | 15.8 |
| *Australopithecus* | Sts 14 | 16.0 | 18.4 | 16.5 | 21.1 | 18.1 | 16.6 |
|  | StW 431 | 24.1 | 36.5 | - | - | 22.4 | 22.5 |
|  | Sediba MH2 | 20.1 | 28.6 | 20.7 | 31.9 | 21.2 | 20.6 |
|  | Stw-H8/H41 | 21.6 | 34.2 | - | - | - | - |
| PS3 | | | | | | | |
| Modern human | Adult  N = 73 | 33.5 ± 3.3 | 45.8 ± 4.1 | 33.1 ± 3.0 | 49.5 ± 4.6 | 25.8 ± 1.7 | 25.7 ± 1.5 |
|  | Juvenile  N = 30 | 26.1 ± 4.9 | 36.3 ± 6.6 | 23.7 ± 3.6 | 36.2 ± 5.2 | 19.7 ± 5.1 | 19.3 ± 5.4 |
| Pan | Adult  N =12 | 27.5 ± 7.1 | 37.3 ± 8.9 | 28.5 ± 7.4 | 39.1 ± 8.7 | 21.9± 4.3 | 24.4 ± 5.2 |
|  | Juvenile  N = 1 | 15.8 | 20.5 | 15.7 | 24.2 | 14 | 15.2 |
|  | Infant  N = 1 | 11.8 | 19.9 | 12.5 | 20.4 | 10.9 | 11 |
| Neandertal | Kebara 2 | 39.5 | 47.19 | 38.44 | 52.87 | 23.27 | 28.01 |
|  | Shanidar 3 | 37.59 | 51.8 | 36.01 | 51.86 | 26.51 | 28.93 |
|  | La Chapelle aux Saints | 36.8 | 50.4 | 36.7 | 53.5 | 24.7 | 27.2 |
| H. erectus | Nariokotome | 27.3 | 38.6 | 28 | 37 | 19 | 17.1 |
| *Australopithecus* | Sts 14 | 18.4 | 26.2 | 17.8 | 27 | 19.4 | 19.1 |
|  | StW 431 | 24.4 | 36.2 | - | - | 22.8 | 23.9 |
|  | A.L. 288-1 aa/ak/al | 16.5 | 29.9 | - | - | 19.9 | 21.7 |
|  | Sediba MH1 | 19.6 | 30.9 | 20.7 | 30.4 | 13.5 | 13.8 |
|  | Stw-H8/H41 | 23.2 | 32.2 | - | - | 20 | 21.5 |
|  | StW 656 | 22.8 | 34.4 | 24.7 | 37 | 23.4 | 25.2 |

**Supplementary 2, Table 3.** List of specimens studied in the current research.

| Group | Age | N. | Vertebra | Osteological material/CT scan/ surface scan | Source of material | Ref. |
| --- | --- | --- | --- | --- | --- | --- |
| Extant hominoids | | | | | | |
| Modern humans | Adult | 73 | PS1-PS3 | Osteological material | Tel Aviv University, Medical School Osteological Collection.  Duckworth collection, Cambridge University. Osteological collection, Musee de l' Homme, Paris France. | ^61,62^ |
|  | Subadult and juvenile | 30 | PS1-PS3 | Osteological material (4), CT scan (26) | Bar Ilan University, Medical School Osteological Collection.  Radiological collection, Sheba Medical Center. | Current study. |
| Pan | Adult | 12 | PS1-PS3 | Osteological material (1), CT scan (11) | Morphosource. Kupri.  Tel Aviv University, Medical School Osteological Collection. | ^61^  Current study. |
|  | Subadult and juvenile | 2 | PS1-PS3 | CT scan | Morphosource. Kupri. ref | Current study. |
| Extinct Hominins | | | | | | |
| Neandertals | | | | | | |
| Kebara 2 | Adult | 1 | PS1-PS3 | Original osteological material | Tel Aviv University, Medical School Osteological Collection. | ^63^ |
| Shanidar 3 | Adult | 1 | PS1-PS3 | Original osteological material | Osteological collection, Smithsonian institute, Washington. | ^63^ |
| La Chapelle aux Saints | Adult | 1 |  | Original osteological material | Osteological collection, Musee de l'Homme, Paris France. | ^63^ |
| H. erectus | | | | | | |
| Nariokotome | Subadult | 1 | PS1-PS3 | High quality cast | Ono Academic College, Osteological Collection. | Current study |
| Australopithecus | | | | | | |
| Sts 14 | Subadult | 1 | PS1-PS3 | CT scan | Bar Ilan University, Medical School Osteological Collection. | Current study |
| Stw 431 | Adult | 1 | PS1-PS3 | High quality cast | Tel Aviv University, Medical School Osteological Collection. | Current study |
| SK 853/3981 | Adult | 1 |  | High quality cast | Tel Aviv University, Medical School Osteological Collection. | Current study |
| Sediba MH1 |  | 1 |  | Surface scan | Morphosource | Current study |
| Sediba MH2 |  | 1 |  | Surface scan | Morphosource | Current study |
| AL 288-1 (Lucy) | Adult | 1 | PS3 | High quality cast | Tel Aviv University, Medical School Osteological Collection. | Current study |
| Stw-H8/H41 | Adult | 1 | PS2, PS3 | Original osteological material |  | ^64^ |
| StW 656, StW 600 | Adult | 1 | PS1, PS3 | Original osteological material |  | ^65^ |

Supplementary 2, Table 4. Ratios/Indexes of vertebral dimensions for modern humans, chimpanzees, extinct hominins and UB 10749 for three spinal vertebrae: PS1, PS2, and PS3.

| Group | Ratio  Specimens | Superior length/ superior width ratio | Inferior length/ inferior width ratio | Posterior height/ anterior height ratio | Superior length/ posterior height ratio |
| --- | --- | --- | --- | --- | --- |
|  | UB 10749 | 0.77 | 0.72 | 0.88 | 1.65 |
| **PS1** | | | | | |
| Modern human | Adult  N = 73 | 0.68 ± 0.04 | 0.66 ± 0.04 | 0.81 ± 0.06 | 1.57 ± 0.15 |
|  | Juvenile  N = 30 | 0.65 ± 0.04 | 0.62 ± 0.04 | 0.82 ± 0.06 | 1.62 ± 0.20 |
| Pan | Adult  N =12 | 0.69 ± 0.05 | 0.66 ± 0.07 | 1.01 ± 0.07 | 1.14 ± 0.15 |
|  | Juvenile  N = 1 | 0.67 | 0.63 | 1.07 | 1.15 |
|  | Infant  N = 1 | 0.61 | 0.59 | 0.95 | 1.14 |
| Neandertal | Kebara 2 | 0.67 | 0.66 | 0.81 | 1.49 |
|  | Shanidar 3 | 0.70 | 0.69 | 0.78 | 1.70 |
|  | La Chapelle aux Saints | 0.65 | 0.70 | 0.89 | 1.51 |
| H. erectus | Nariokotome | 0.66 | 0.65 | 0.76 | 1.85 |
| *Australopithecus* | Sts 14 | 0.69 | 0.65 | 0.89 | 1.13 |
|  | Stw 431 | 0.62 | - | 0.89 | 1.16 |
|  | SK 853/3981 | 0.60 | - | 0.89 | 1.24 |
|  | Sediba MH2 | 0.66 | 0.70 | 0.83 | 1.28 |
|  | StW 600 | 0.59 | 0.67 | 0.88 | 1.21 |
| PS2 | | | | | |
| Modern human | Adult  N = 73 | 0.70 ± 0.04 | 0.65 ± 0.04 | 0.92 ± 0.06 | 1.39 ± 0.14 |
|  | Juvenile  N = 30 | 0.69 ± 0.04 | 0.65 ± 0.04 | 0.93 ± 0.05 | 1.44 ± 0.14 |
| Pan | Adult  N =12 | 0.72 ± 0.06 | 0.71 ± 0.05 | 1.06 ± 0.05 | 1.11 ± 0.12 |
|  | Juvenile  N = 1 | 0.74 | 0.62 | 0.99 | 1.15 |
|  | Infant  N = 1 | 0.62 | 0.59 | 0.95 | 1.07 |
| Neandertal | Kebara 2 | 0.74 | 0.73 | 1.05 | 1.37 |
|  | Shanidar 3 | 0.67 | 0.67 | 0.99 | 1.22 |
|  | La Chapelle aux Saints | 0.69 | 0.68 | 0.98 | 1.51 |
| H. erectus | Nariokotome | 0.69 | 0.69 | 0.82 | 1.74 |
| *Australopithecus* | Sts 14 | 0.86* | 0.078* | 0.91* | 0.96* |
|  | StW 431 | 0.66 | - | 0.99 | 1.07 |
|  | Sediba MH2 | 0.70 | 0.65 | 0.97 | 0.97 |
|  | Stw-H8 | 0.63 | - | - | - |
| PS3 | | | | | |
| Modern human | Adult  N = 73 | 0.73 ± 0.05 | 0.67 ± 0.04 | 1.00 ± 0.03 | 1.30 ± 0.08 |
|  | Juvenile  N = 30 | 0.72 ± 0.05 | 0.66 ± 0.03 | 0.98 ± 0.05 | 1.39 ± 0.16 |
| Pan | Adult  N =12 | 0.74 ± 0.08 | 0.73 ± 0.08 | 1.11 ± 0.07 | 1.12 ± 0.08 |
|  | Juvenile  N = 1 | 0.77 | 0.65 | 1.08 | 1.04 |
|  | Infant  N = 1 | 0.59 | 0.61 | 1.01 | 1.18 |
| Neandertal | Kebara 2 | 0.84 | 0.72 | 1.20 | 1.41 |
|  | Shanidar 3 | 0.73 | 0.69 | 1.09 | 1.29 |
|  | La Chapelle aux Saints |  |  | 1.10 | 1.35 |
| H. erectus | Nariokotome | 0.71 | 0.76 | 0.90 | 1.59 |
| *Australopithecus* | Sts 14 | 0.70 | 0.66 | 0.98 | 0.96 |
|  | StW 431 | 0.67 |  | 1.04 | 1.02 |
|  | A.L. 288-1 aa/ak/al | 0.55 |  | 1.09 | 0.76 |
|  | Sediba MH1 | 0.69 | - | 1.02 | 1.41 |
|  | Stw-H8 | 0.63 | - | 1.07 | 1.08 |
|  | StW 656 | 0.66 | 0.67 | 1.07 | 0.90 |

#### Supplementary 2, Table 5. Vertebral body size (PS1-PS3) of modern human children and adults with known age. Data was acquired from CT scans.

|  | PS1 | | PS2 | | PS3 | |
| --- | --- | --- | --- | --- | --- | --- |
| Age (year.month) | Superior Length | superior Width | Superior Length | superior Width | Superior Length | superior Width |
| 0.2 | 10.8 | 16.9 | 10.3 | 18.5 | 10.6 | 18.8 |
| 1.0 | 13.7 | 23 | 14.6 | 22.9 | 15.1 | 22.6 |
| 1.5 | 12.8 | 25.9 | 13.3 | 26.3 | 13 | 24.6 |
| 1.75 | 16 | 29.5 | 16.4 | 27.8 | 17 | 28.7 |
| 2.0 | 14.4 | 25.2 | 14.4 | 25.7 | 14.4 | 25.5 |
| 2.3 | 15.8 | 25.9 | 15.5 | 25.3 | 14.4 | 24.2 |
| 2.6 | 16.2 | 26.5 | 15.2 | 25.7 | 15.1 | 25.8 |
| 3.11 | 19.5 | 32.1 | 19.3 | 31.3 | 19.4 | 29.1 |
| 3.3 | 21.8 | 31 | 21.6 | 31.4 | 21.6 | 29.3 |
| 3.7 | 17.8 | 29.1 | 18.3 | 28.5 | 18.1 | 27.7 |
| 4.7 | 20.8 | 31.6 | 19.9 | 30 | 19.4 | 30 |
| 4.9 | 19.6 | 30.2 | 20.3 | 28.6 | 21.4 | 29.3 |
| 5.5 | 22.8 | 33 | 22.4 | 34.2 | 21.6 | 31.2 |
| 5.8 | 20.6 | 31.7 | 19.6 | 29.8 | 18.5 | 28.9 |
| 6.0 | 22.4 | 33.4 | 24.5 | 35.4 | 24.2 | 32.3 |
| 6.6 | 21.2 | 31.5 | 21.6 | 29.4 | 20.9 | 29.1 |
| 6.7 | 24.4 | 38.7 | 23.6 | 33 | 24 | 31.3 |
| 6.9 | 22.7 | 35.3 | 21.6 | 33.1 | 20.6 | 31.8 |
| 7.11 | 26.2 | 36 | 24.9 | 34.5 | 24.5 | 30.2 |
| 7.3 | 23.6 | 34.6 | 21.5 | 32.3 | 20.1 | 31.6 |
| 8.11 | 24.8 | 36.3 | 24.8 | 34.8 | 23.9 | 33.9 |
| 8.4 | 23.2 | 34.4 | 22.4 | 32.4 | 21.2 | 30 |
| 9.1 | 22.8 | 36.8 | 21.8 | 35.5 | 21.9 | 33.7 |
| 9.11 | 24.7 | 40.8 | 23.9 | 38.3 | 23.8 | 35.4 |
| 9.8 | 24.6 | 40.2 | 25.9 | 39.8 | 24.5 | 35.3 |
| 10.1 | 28.1 | 43.6 | 27.2 | 42.7 | 27.7 | 39.7 |
| 10.4 | 26.9 | 40.7 | 25.9 | 39.8 | 25.5 | 37.6 |
| 11.1 | 26.8 | 45.9 | 27.8 | 42.6 | 27.8 | 40.1 |
| 11.11 | 29.8 | 43 | 29.5 | 42.1 | 28.1 | 39.1 |
| 11.3 | 27.6 | 44.69 | 27.3 | 44.2 | 27.3 | 39.8 |
| 12.2 | 27.5 | 42.2 | 26.12 | 38.3 | 27.8 | 36.5 |
| 12.8 | 30.6 | 49.2 | 28.8 | 45.7 | 28.7 | 43.7 |
| 13.11 | 33.3 | 51 | 33.1 | 48.4 | 32.2 | 45 |
| 13.2 | 28.1 | 45.2 | 27.1 | 44.5 | 28.3 | 42.5 |
| 13.9 | 32.1 | 47.1 | 30.3 | 43.1 | 28.9 | 41.7 |
| 14.11 | 33 | 48.7 | 33.5 | 49 | 31.4 | 45.2 |
| 14.7 | 37.8 | 53.2 | 36.4 | 50.8 | 37.7 | 49.9 |
| 15.11 | 31.7 | 46.2 | 33.3 | 46.2 | 34.4 | 44.9 |
| 15.2 | 34.6 | 49.8 | 31 | 45 | 30.3 | 43.2 |
| 15.5 | 28.4 | 45.3 | 28.7 | 37 | 29 | 44.1 |
| 16.0 | 30.6 | 45.9 | 28.9 | 44.1 | 28.1 | 42.2 |
| 16.2 | 33.9 | 54.1 | 32.1 | 51 | 34.2 | 49.2 |
| 17 | 27.2 | 46.7 | 26.5 | 43 | 25.1 | 42.5 |
| 17.11 | 31.3 | 47 | 30.5 | 46.7 | 30.1 | 43.5 |
| 17.8 | 31.7 | 45.1 | 31.1 | 42 | 30.6 | 38.7 |
| 18.0 | 34 | 54.6 | 33.6 | 51.3 | 34.7 | 50.4 |
| 18.11 | 32.8 | 48.9 | 31.7 | 46.6 | 32.9 | 43.7 |
| 19.0 | 33.9 | 52.6 | 32.1 | 48.3 | 32.6 | 44.7 |
| 19.8 | 29.7 | 46.5 | 30.3 | 45.9 | 28.7 | 41.8 |
| 19.9 | 39.9 | 57.6 | 39.6 | 55.9 | 39.1 | 54 |

####

#### Supplementary 2, Table 6. Height estimation at death for UB 10749. Calculations are based on Chinese^66^ and German^67^ populations.

| Measurement | linear regression model | SE | R | UB 10749 Length (mm) | **Calculated height for UB 10749 (cm)** |
| --- | --- | --- | --- | --- | --- |
| The equations are based on Chinese population aged 20-45 years^66^ | | | | | |
| PS1 | | | | | |
| Vertebral anterior height (mm) | | | | 22 |  |
| Male | Y=137.701+1.112X | 5.470 | 0.408 |  | **162.164** |
| Female | Y=128.244+1.146X | 4.624 | 0.493 |  | **153.45** |
| Vertebral posterior height (mm) | | | | 20 |  |
| Male | Y=133.49+1.337X | 5.248 | 0.483 |  | **160.23** |
| Female | Y=120.619+1.575X | 3.900 | 0.680 |  | **152.119** |
| PS2 | | | | | |
| Vertebral anterior height (mm) | | | | 22 |  |
| Male | Y=125.314+1.585X | 5.805 | 0.529 |  | **160.184** |
| Female | Y=116.636+1.572X | 3.873 | 0.685 |  | **151.22** |
| Vertebral posterior height (mm) | | | | 20 |  |
| Male | Y=121.49+1.698X | 4.931 | 0.568 |  | **155.45** |
| Female | Y=115.637+1.645X | 3.739 | 0.711 |  | **148.54** |
| PS3 | | | | | |
| Vertebral anterior height (mm) | | | | 22 |  |
| Male | Y=113.27+1.983X | 4.592 | 0.642 |  | **156.896** |
| Female | Y=115.24+1.552X | 4.033 | 0.651 |  | **149.38** |
| Vertebral posterior height (mm) | | | | 20 |  |
| Male | Y=111.32+1.932X | 4.371 | 0.684 |  | **149.96** |
| Female | Y=116.539+1.494X | 3.956 | 0.668 |  | **146.419** |
| The equations are based on PS3 of German population, aged 20-64 years^67^ | | | | | |
| Vertebral anterior height | Y=153.6+8.2X | 6.7 | 0.07 | 22 | **171.64** |
| Vertebral posterior height | Y=122.3+18.5X | 6.0 | 0.27 | 20 | **159.3** |
| **Average estimated height for UB 10749** |  |  |  |  | **155.45** |

**Supplementary 2, Table 7.** Weight estimation at death for UB 10749, assuming an age of death of 6-12 years, and a height of 155 cm.

| Based on height/age | Formula | Weight estimation for UB 10749 (kg) |
| --- | --- | --- |
| Height | Based on adult population^68^ | 56.67 ± 6.71 |
| Height | Based on children^69^ | 45 |
| Age | *The advanced pediatric life support (APLS) formula: Weight = 2x(age+4)^70^ | 20 – 32 |
| Age | The Luscombe formula:  weight = 3x(age)+7^70^ | 25 – 43 |
| Age | 50th centile in growth charts^69^ | 20 – 41 |

*****This formula is known to underestimate weight for children post World War 2.

Supplementary 2, Table 8. Predicted adult size (height and weight) for UB 10749 based on growth charts of chimpanzees and modern humans.

| Growth charts of *Pan*/Modern humans | Age (years) | Height (cm) | % of adult size | Prediction of adult height for UB 10749 (cm) | Weight (kg) | % of adult size | Prediction of adult weight for UB 10749 (kg) |
| --- | --- | --- | --- | --- | --- | --- | --- |
| *Pan troglodytes*^71^ | 6 | 117 | 80% | 192 | 27 | 50% | 91-101 |
|  | 8 | 130 | 89% | 172 | 37 | 67% | 66-74 |
|  | 10 | 140 | 96% | 160 | 48 | 87% | 51-57 |
|  | 12 | 145 | 100% | 155 | 55 | 100% | 45-50 |
| Modern humans, American boys^69^ | 6 | 115 | 64% | 240 | 21 | 30% | 150-166 |
|  | 8 | 128 | 71% | 215 | 26 | 37% | 121-134 |
|  | 10 | 139 | 78% | 198 | 32 | 45% | 98-109 |
|  | 12 | 148 | 83% | 186 | 40 | 57% | 78-87 |
| Modern humans, American girls^69^ | 6 | 115 | 70% | 221 | 20 | 34% | 130-145 |
|  | 8 | 128 | 78% | 198 | 26 | 45% | 100-111 |
|  | 10 | 138 | 84% | 184 | 33 | 56% | 79-87 |
|  | 12 | 151 | 92% | 168 | 42 | 72% | 62-69 |
| Modern humans, Sudanese boys^72^ | 6 | 107 | 62% | 247 | 17 | 29% | 156-173 |
|  | 8 | 122 | 71% | 217 | 21 | 35% | 126-140 |
|  | 10 | 130 | 76% | 203 | 24 | 40% | 110-122 |
|  | 12 | 137 | 80% | 193 | 29 | 49% | 91-101 |
| Modern humans, Sudanese girls^72^ | 6 | 107 | 66% | 233 | 17 | 31% | 142-158 |
|  | 8 | 117 | 72% | 213 | 20 | 37% | 121-135 |
|  | 10 | 127 | 78% | 196 | 25 | 46% | 97-108 |
|  | 12 | 140 | 86% | 178 | 30 | 55% | 81-90 |

#### Supplementary 2. Figures:

#### Supplementary 2, Fig. 1. Vertebral body wedging (posterior/anterior height ratio) of UB 10749 and adult modern humans (for three lumbar vertebrae PS1-PS3). Note that the ratio of UB 10749 is most similar to that of PS2.


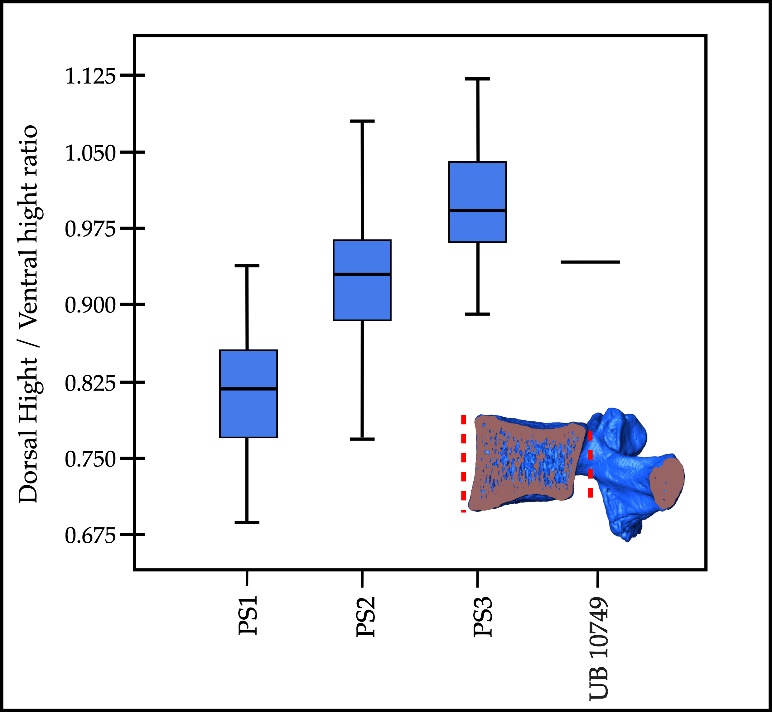


**Supplementary 2, Fig. 2.** Pedicle ossification level across ages.


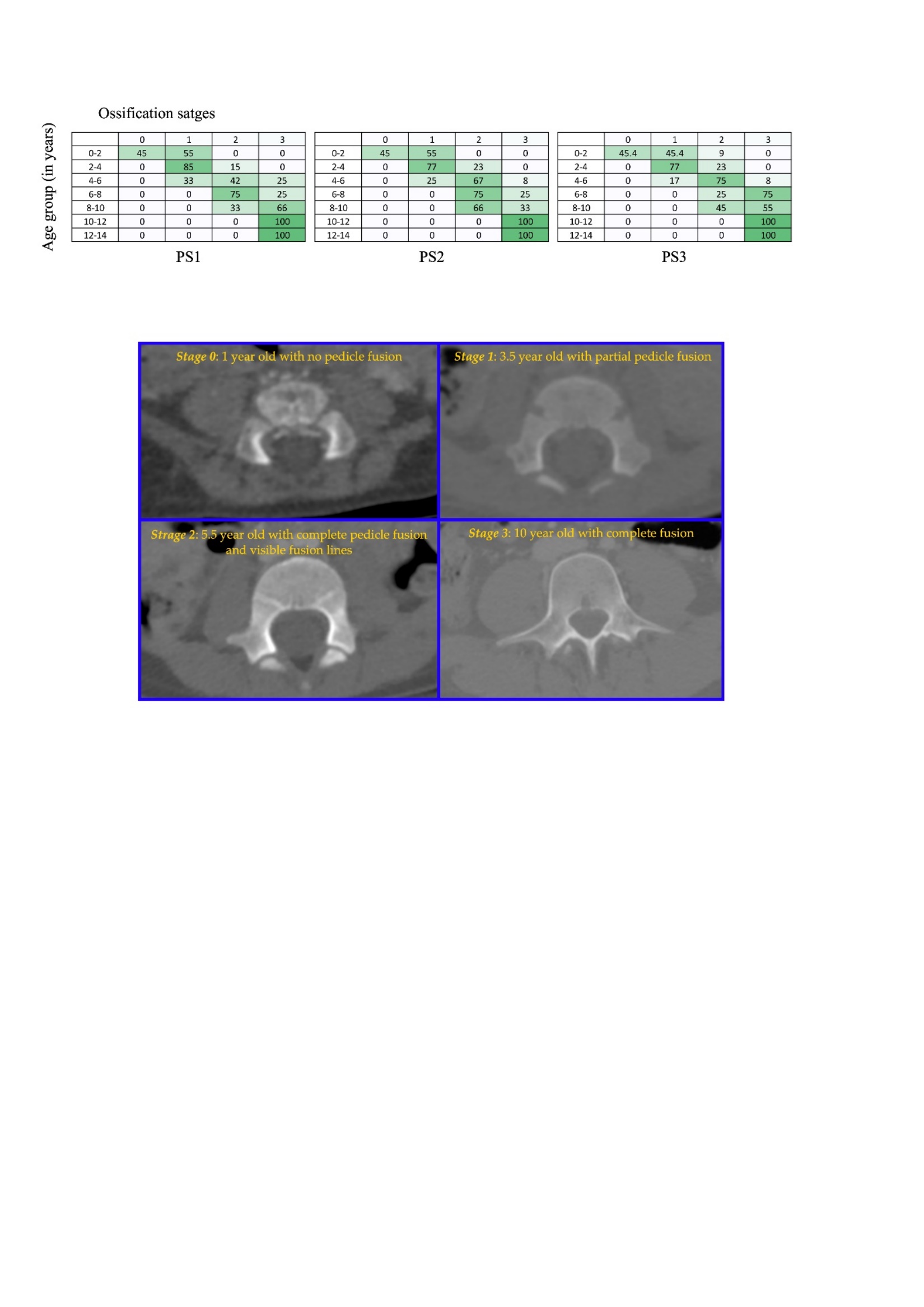
Percentage of pedicle ossification at different age groups for PS1, PS2 and PS3. Each age group includes 8-10 individuals. N= 65.

Level of ossification was determined as:

0: No ossification. A gap is present between vertebral body and pedicle.

1: Partial ossification. Portion of the epiphysis is ossified, but not the entire gap.

2: Full ossification. A fusion line is visible between vertebral body and pedicle.

3: Full ossification. No fusion line is apparent.

#### Supplementary 2, Fig. 3. Anterolateral view of UB 10749 showing the location of the landmarks and semi-landmarks used in the study.

####
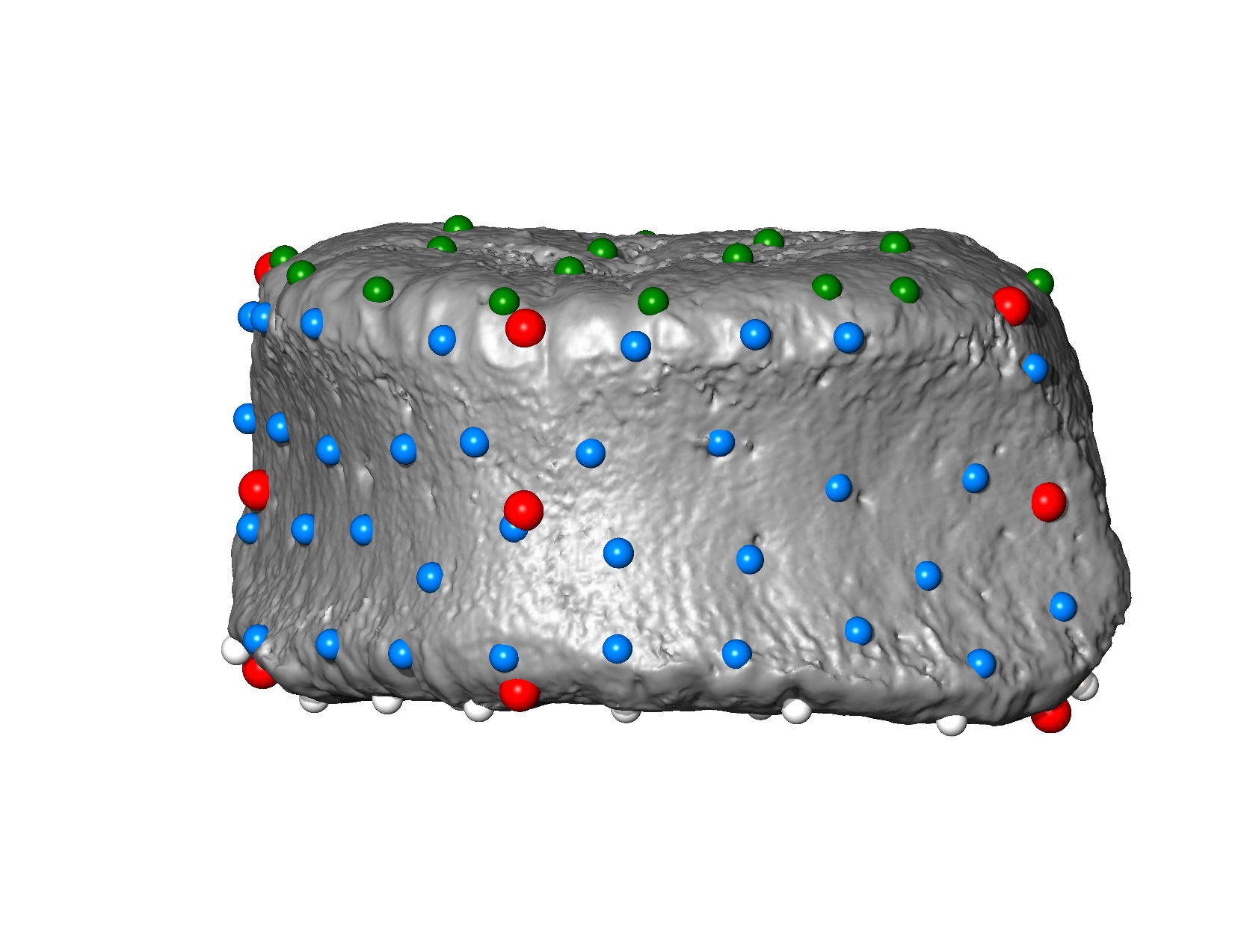


#### Supplementary 2, Fig. 4. Linear discriminant analysis of age groups, of modern humans and UB 10749. UB 10749 falls within the 6-10 years old age group.


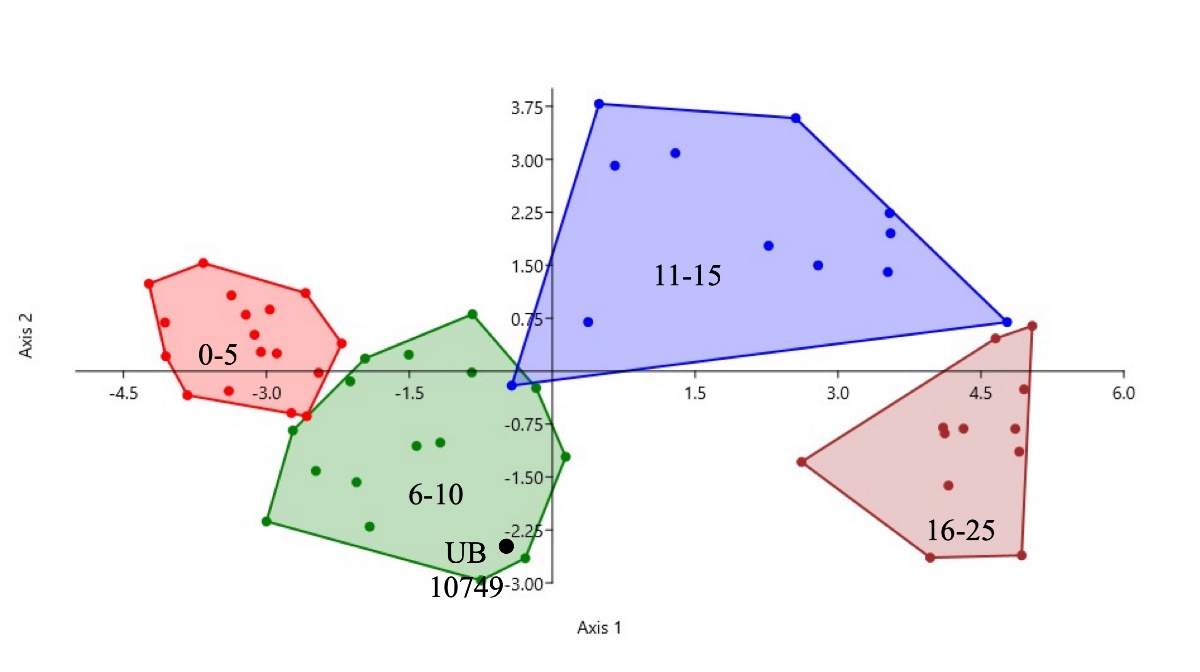


#### Supplementary 2, Fig. 5. Reconstruction of *Australopithecus africanu*s STS 14 (PS1-3).

STS 14 was discovered in Sterkfontein, South Africa, in 1947 and described by Robert Broom and John Robinson. It is assigned to adult or subadult *Australopithecus africanus*^73^. All the lumbar vertebrae of this specimen were recovered. PS1, originally named STS 14a, is relatively complete, and did not need any reconstruction of the vertebral body. PS2, originally named STS 14b, is broken and missing the right anterior margin of the vertebral body. Using AVIZO 9.3 ([www.fei.com](http://www.fei.com)), the complete left side was mirror onto the right side and aligned through superimposing similar landmarks on both parts. A small anterior fragment was still absent from the superior and inferior rim of the vertebra. These were reconstructed using their corresponding parts on the upper and lower adjacent vertebrae. PS3, originally named STS 14c is broken on the right side. The complete left side was mirrored imaged into the right side using similar landmarks superimposition.


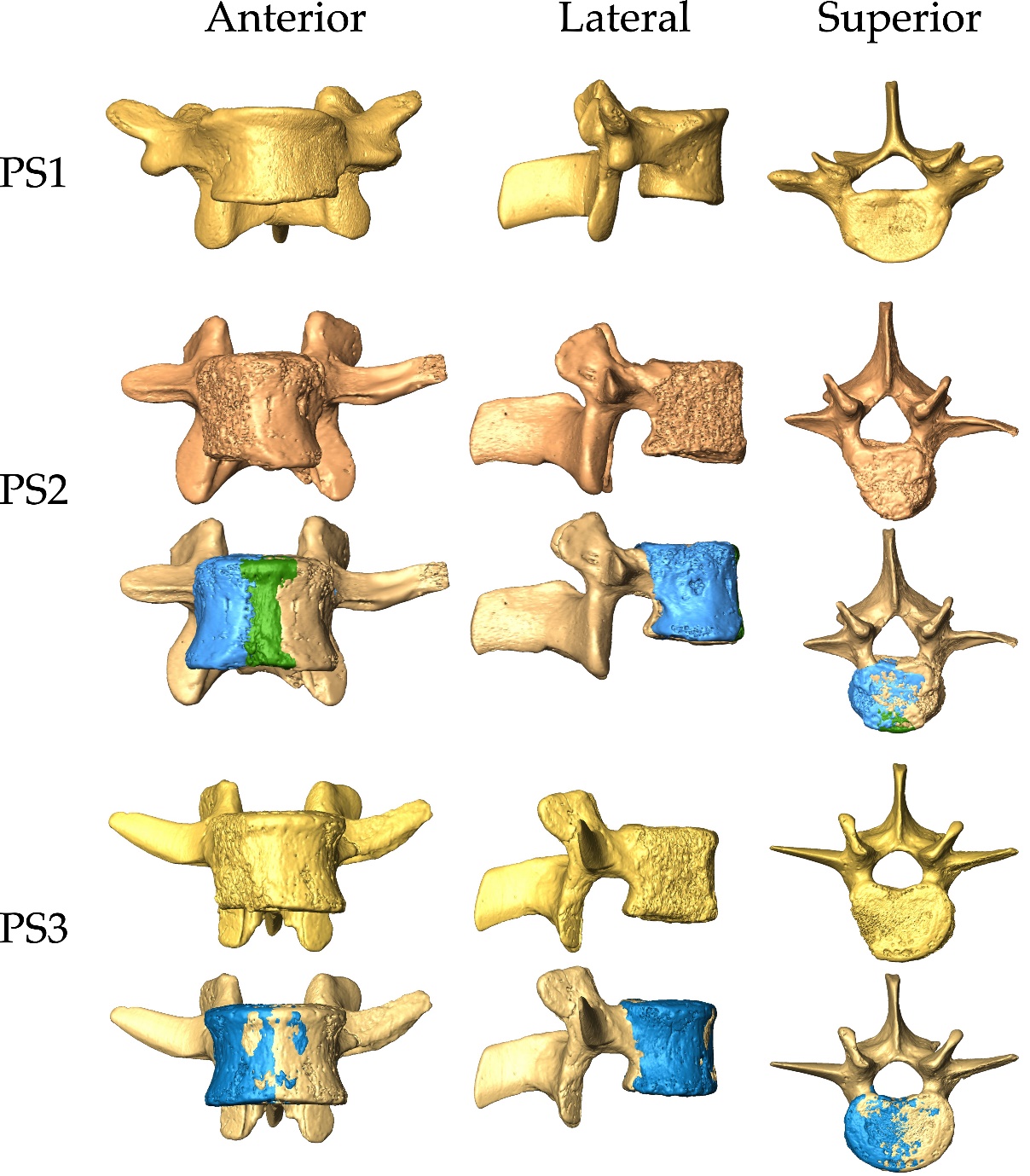


1. **References.**
2. Picard, L., & Baida, U. Geological report on the lower Pleistocene deposits of the ‘Ubeidiya excavations. Jerusalem: *Isr. Acad. Sci. Human.* (1966).
3. Picard, L., & Baida, U. Stratigraphic position of the ‘Ubeidiya formation. Jerusalem: *Isr. Acad. Sci. Human.* (1966).
4. Bar-Yosef, O., & Tchernov, E. On the Palaeo-ecological History of the Site of ʻUbeidiya. Jerusalem: *Isr. Acad. Sci. Human.* (1972).
5. Bar-Yosef, O., & Goren-Inbar, N. The lithic assemblages of ‘Ubeidiya, a lower paleolithic site in the Jordan valley. Jerusalem: *The Institute of Archaeology, The Hebrew University of Jerusalem*. (1993).
6. Stekelis, M. Archeological Excavations at ‘Ubeidiya. 1960-1963. *Jerusalem: Isr. Acad. Sci.* (1966).
7. Matmon, A., Zilberman, E., Enzel, Y., & Bar-Yosef, O. Landscape evolution along the Dead Sea fault and its margins. Quaternary of the Levant, edited by: Enzel, Y. and Bar-Yosef, O., Cambridge University Press, Cambridge, 17-30. (2017).
8. Horowitz, A. The quaternary of Israel. New York: Academic Press. (1979).
9. Davis, M., Matmon, A., Fink, D., Ron, H., & Niedermann, S. Dating Pliocene lacustrine sediments in the central Jordan valley, Israel? Implications for cosmogenic burial dating. *Earth. Planet. Sci. Lett*. **305**, 317-327 (2011).
10. Tchernov, E. The early Pleistocene molluscs of'Erq el-Aḥmar. *Isr. Acad. Sci. Human.* (1975).
11. Picard, L. Structure and evolution of palestine. *Bulletin of the Geological Department, Hebrew University*, **4**, 1-134 (1943).
12. Tchernov, E. (Ed.). Les mammifères du pléistocène inférieur de la vallée du jourdain a oubéidiyeh. Paris: Association Paléorient. (1986).
13. Verosub, K., & Tchernov, E. Resultats préliminaires de l’étude magnétostratigraphique d’une séquence sédimentaire à l’industrie humaine en israël. In B. Vandermeersch (Ed.), Les Premiers Peuplements de l’Europe (pp. 237-242). Paris: C.N.R.S. (1991).
14. Sagi, A. (2005). Magnetostratigraphy of ‘ubeidiya formation, northern dead sea transform, israel. Department of Earth Sciences.
15. Sagi, A., Belmaker, M., Ron, H., Enzel, Y., Agnon, A., & Bar Yosef, O. Paleomagnetic dating of `Ubeidiya formation. In S. Abramovich (Ed.), Abstracts of the Israel Geological Society Annual Meeting 2005 (p. 101). *Isr. Geol. Societ.* (2005).
16. Rink, W.J., Bartoll J., Schwarcz H.P., Shane P., & Bar-Yosef O. Testing the reliability of ESR dating of optically exposed buried quartz sediments. *Radiat. Meas*. **42**, 1618-1626 (2007).
17. Haas, G. Preliminary remarks on the early Quarternary faunal assemblage from Tel Ubeidiya, Jordan Valley. *S. Afr. J. Sci.* **59**, 73-76‏ (1963).
18. Belmaker, M. Community structure through time:'Ubeidiya, a Lower Pleistocene site as a case study. *Hebrew University of Jerusalem*.‏ (2006).
19. Martinez-Navarro, B., Belmaker, M., & Bar-Yosef, O. The large carnivores from ‘Ubeidiya (early Pleistocene, Israel): biochronological and biogeographical implications. *J. Hum. Evol.* **56**, 514-524‏ (2009).
20. Guérin, C. (1982). Première biozonation du pléistocène européen, principal résultat biostratigraphique de l’étude des rhinocerotidae (mammalia, perissodactyla) du miocène terminal au pléistocène supérieur d’europe occidentale. Geobios, 15(4), 593-598.
21. Caloi, L., & Palombo, M. R. Biochronology of large mammals in the early and middle pleistocene on the italian peninsula. *Hystrix*. **9**, 3-12 (1997).
22. Bar-Yosef, O., & M. Belmaker. "Ubeidiya." In Quaternary of the Levant: Environments, Climate Change, and Humans. Eds.: Y. Enzel, p. 179 (2017).
23. Gaudzinski, S. Subsistence patterns of Early Pleistocene hominids in the Levant—taphonomic evidence from the'Ubeidiya Formation (Israel). *J. Archaeol. Sci.* **31**, 65-75‏ (2004).
24. Mellado, José M., et al. MDCT of variations and anomalies of the neural arch and its processes: part 1—pedicles, pars interarticularis, laminae, and spinous process. *Am. J. Roentgenol.* **197**, W104-W113‏ (2011).
25. Rajwani, T., et al. MRI characteristics of the neurocentral synchondrosis. *Pediatr. Radiol.* **32**, 811-816‏ (2002).
26. Zhang, H., Sucato, D. J., Nurenberg, P., & McClung, A. Morphometric analysis of neurocentral synchondrosis using magnetic resonance imaging in the normal skeletally immature spine. *Spine*. **35**, 76-82‏ (2010).
27. Lewis, C. P., Lavy, C. B. D., & Harrison, W. J. Delay in skeletal maturity in Malawian children. *J. Bone Joint Surg. Br.* **84**, 732-734‏ (2002).
28. Artal, D., Rosenberg, R. J., & Cronin, E. B. Delayed epiphyseal closure seen on Tc-99m methylene disphosphonate bone scan in association with hypopituitarism. *Clin. Nucl. Med.* **34**, 310-311‏ (2009).
29. Cotten, A. et al. Persistence of the notochordal canal: plain film and CT findings. *Neuroradiology* **37**, 308-310‏ (1995).
30. Christopherson, L. R., Rabin, B. M., Hallam, D. K., & Russell, E. J. Persistence of the notochordal canal: MR and plain film appearance. *Am. J. Neuroradiol.* **20**, 33-36‏ (1999).
31. Taylor, J. R. Persistence of the notochordal canal in vertebrae. *J. Anat.* **111**, 211‏ (1972).
32. Postma, A. V., et al. Mutations in the T (brachyury) gene cause a novel syndrome consisting of sacral agenesis, abnormal ossification of the vertebral bodies and a persistent notochordal canal. *J. Med. Genet.* **51,** 90-97 (2014).‏
33. Martorell, R., Yarbrough, C., Klein, R. E., & Lechtig, A. Malnutrition, body size, and skeletal maturation: interrelationships and implications for catch-up growth. *Hum. Biol.* **51**, 371-389‏ (1979).
34. Martelli, S. A. The modern and fossil hominoid spinal ontogeny. In *Spinal Evolution* (pp. 247-281) edited by: Been, E., Gomez-Olivencia, A., Kramer, P. Springer, Cham.‏ (2019).
35. Rosas, Antonio, et al. The growth pattern of Neandertals, reconstructed from a juvenile skeleton from El Sidrón (Spain). *Science.* **357,** 1282-1287 (2017).
36. ‏ Cunningham, D. L., Graves, R. R., Wescott, D. J., & McCarthy, R. C. The effect of ontogeny on estimates of KNM-WT 15000's adult body size. *J. Hum. Evol.* **121**, 119-127‏ (2018).
37. Gabunia, L. *et al.* Earliest Pleistocene hominid cranial remains from Dmanisi, Republic of Georgia: taxonomy, geological setting, and age. *Science*. **288**, 1019-1025 (2000).
38. Lordkipanidze, D. et al. Postcranial evidence from early Homo from Dmanisi, Georgia. *Nature*. **449**, 305-310 (2007).
39. Rightmire, G. P., Lordkipanidze, D., & Vekua, A. Anatomical descriptions, comparative studies and evolutionary significance of the hominin skulls from Dmanisi, Republic of Georgia. *J. Hum. Evol.* **50,** 115-141‏ (2006).
40. Rightmire, G. P., Van Arsdale, A. P., & Lordkipanidze, D. Variation in the mandibles from Dmanisi, Georgia. *J. Hum. Evol.* **54**, 904-908‏ (2008).
41. Ferring, R. *et al.* Earliest human occupations at Dmanisi (Georgian Caucasus) dated to 1.85–1.78 Ma. *Proc. Natl. Acad. Sci*. **108**, 10432-10436‏ (2011).
42. Simpson, S. W. *et al.* A female Homo erectus pelvis from Gona, Ethiopia. *Science* **322**, 1089-1092 (2008).
43. Swisher, C. C., Curtis, G. H., Jacob, T., Getty, A. G., & Suprijo, A. Age of the earliest known hominids in Java, Indonesia. *Science* **263**, 1118-1121 (1994).
44. Huffman, O. F. Geologic context and age of the Perning/Mojokerto Homo erectus, East Java. *J. Hum. Evol.* **40**, 353-362‏ (2001).
45. Larick, R., *et al.* Early Pleistocene 40Ar/39Ar ages for Bapang formation hominins, central Jawa, Indonesia. *Proc. Natl. Acad. Sci.***98**, 4866-4871‏ (2001).
46. Zaim, Y. Geological evidence for the earliest appearance of hominins in Indonesia. *Out of Africa I*. Springer, Dordrecht, 97-110‏ (2010).
47. Gibert, G., Ribot, F., Ferrández, C., Martínez, B., & Caporicci, R. Caracteristicas diferenciales entre el fragmento de craneo de Homo sp. de Venta Micena (Orce, Granada) y los Equidos. *Estud. Geol.* **45**, 121-138‏ (1989).
48. Gibert, J., & Jiménez, C. Investigations into cut-marks on fossil bones of Lower Pleistocene age from Venta Micena (Orce, Granada province, Spain). *Hum. Evol*. **6**, 117-127‏ (1991).
49. Gibert, J. *et al*. Hominid status of the Orce cranial fragment reasserted. *J. Hum. Evol.* **30**, 203-217 (1998).
50. Feibel, C. S., Brown, F. H., & McDougall, I. Stratigraphic context of fossil hominids from the Omo Group deposits: northern Turkana Basin, Kenya and Ethiopia. *Am. J. Phys.l Anthropol.* **78**, 595-622‏ (1989).
51. Brown, F., Harris, J., Leakey, R., & Walker, A. Early Homo erectus skeleton from west lake Turkana, Kenya. *Nature*, **316**, 788-792‏ (1985).
52. Toro-Moyano, I. *et al.* The oldest human fossil in Europe, from Orce (Spain). *J. Hum. Evol.* **65**, 1-9‏ (2013).
53. Carbonell, E. *et al.* The first hominin of Europe. *Nature*, **452**, 465-469‏ (2008).
54. De Castro, J. B. *et al.* Early Pleistocene human mandible from Sima del Elefante (TE) cave site in Sierra de Atapuerca (Spain): a comparative morphological study. *J. Hum. Evol.* **61**, 12-25‏ (2011).
55. Parés, J. M., *et al.* Matuyama-age lithic tools from the Sima del Elefante site, Atapuerca (northern Spain). *J. Hum. Evol.* **50**, 163-169‏ (2006).
56. Abbate, E. *et al.* A one-million-year-old Homo cranium from the Danakil (Afar) Depression of Eritrea. *Nature*, **393**, 458-460‏ (1998).
57. Zanolli, C. *et al.* The late early pleistocene human dental remains from Uadi Aalad and Mulhuli-Amo (Buia), Eritrean Danakil: macromorphology and microstructure. *J. Hum. Evol.***74**, 96-113‏ (2014).
58. Palmqvist, P., Pérez-Claros, J. A., Gibert, J., & Santamarıa, J. L. Comparative morphometric study of a human phalanx from the lower Pleistocene site at Cueva Victoria (Murcia, Spain), by means of Fourier analysis, shape coordinates of landmarks, principal and relative warps. *J. Archaeol. Sci.* **23**, 95-107‏ (1996).
59. Gibert, L. *et al.* Chronology for the Cueva Victoria fossil site (SE Spain): evidence for early Pleistocene Afro-Iberian dispersals. *J. Hum. Evol.* **90**, 183-197‏ (2016).
60. Carbonell, E. *et al.* Lower Pleistocene hominids and artifacts from Atapuerca-TD6 (Spain). *Science*, **269**, 826-830‏ (1995).
61. De Castro, J. B. *et al.* A hominid from the Lower Pleistocene of Atapuerca, Spain: possible ancestor to Neandertals and modern humans. *Science.* **276**, 1392-1395‏ (1997).
62. Been, E. The anatomy of the lumbar spine of *Homo neanderthalensis* and its phylogenetic and functional implications. *Doctoral dissertation, Tel Aviv University* (2005).
63. Been, E., Peleg, S., Marom, A., & Barash, A. Morphology and function of the lumbar spine of the Kebara 2 Neandertal. *Am. J. Phys. Anthropol.* **142**, 549-557‏ (2010).
64. Gómez-Olivencia, A., Arlegi, M., Barash, A., Stock, J. T., & Been, E. The Neandertal vertebral column 2: The lumbar spine. *J. Hum. Evol.* **106**, 84-101‏ (2017).
65. Sanders, W. J. Comparative morphometric study of the australopithecine vertebral series Stw-H8/H41. *J. Hum. Evol.* **34**, 249-302‏ (1998).
66. Pickering, T. R., Heaton, J. L., Clarke, R. J., & Stratford, D. Hominin vertebrae and upper limb bone fossils from Sterkfontein Caves, South Africa (1998–2003 excavations). *Am. J. Phys. Anthropol.* **168**, 459-480‏ (2019).
67. Zhang, K., Chang, Y. F., Fan, F., & Deng, Z. H. Estimation of stature from radiologic anthropometry of the lumbar vertebral dimensions in Chinese. *Leg. Med.* **17**, 483-488‏ (2015).
68. Klein, A. *et al*. On the relationship between stature and anthropometric measurements of lumbar vertebrae. *Sci. Justice.* **55,** 383-387‏ (2015).
69. Kokong, D. D. *et al*. Estimation of weight in adults from height: a novel option for a quick bedside technique. *Int. J. Emerg. Med.* **11**, 1-9‏ (2018).
70. Kuczmarski, R. J. CDC growth charts: United States (No. 314). *US Department of Health and Human Services, Centers for Disease Control and Prevention, National Center for Health Statistics.*‏ (2000).
71. Luscombe, M. D., Owens, B. D., & Burke, D. Weight estimation in paediatrics: a comparison of the APLS formula and the formula ‘Weight= 3 (age)+ 7’. *Emerg. Med. J.* **28**, 590-593‏ (2011).
72. Ruff, C. B., & Burgess, M. L. How much more would KNM-WT 15000 have grown?. *J. Hum. Evol.* **80**, 74-82‏ (2015).
73. Sukkar, M. Y., Kemm, J. R., Ballal, M. A., & Ahmed, T. S. Growth velocity in children in rural Khartoum, Sudan. *Ann. Hum. Biol.* **7**, 473-479‏ (1980).
74. Broom, R., Robinson, J.T., Schepers, G.W.H. Sterkfontein ape-man, *Plesianthropus*. *Transv. Mus. Mem*. **4**, 1-117 (1950).
